# First draft reference genome and annotation of the alternative oil species *Physaria fendleri*

**DOI:** 10.1093/g3journal/jkae114

**Published:** 2024-05-28

**Authors:** Christopher R Johnston, Patrick J Horn, Ana Paula Alonso

**Affiliations:** BioDiscovery Institute, Department of Biological Sciences, University of North Texas, Denton, TX 76201, USA; BioDiscovery Institute, Department of Biological Sciences, University of North Texas, Denton, TX 76201, USA; BioDiscovery Institute, Department of Biological Sciences, University of North Texas, Denton, TX 76201, USA

**Keywords:** *Physaria fendleri*, lesquerella, genome annotation, Illumina, hybrid assembly

## Abstract

In the wake of increasing demand for renewable energy sources, plant-based sources including alternative oilseeds have come to the forefront of interest. Hydroxy fatty acids (HFAs), produced in a few oilseed species, are important chemical feed stocks for industrial applications. An integrated approach was taken to assemble the first draft genome of the alternative HFA producer *Physaria fendleri* (*n* = 6), an outcrossing species with high heterozygosity. Both de novo transcriptome assemblies and genome assemblies were produced with public and generated sequencing reads. Resulting intermediate assemblies were then scaffolded and patched with multiple data sources, followed by super-scaffolding onto a masked genome of *Camelina laxa* (*n* = 6). Despite a current lack of available resources for the physical mapping of genomic scaffolds of *P. fendleri*, topography of the genome with respect to repeat and gene content was preserved at the scaffold level and not significantly lost via super-scaffolding. Read representation, gene and genome completion statistics, and annotation results illustrated the creation of a functional draft genome and a tool for future research on alternative oil species.

## Introduction

Plant-based specialty oils and fatty acids (FAs) are increasingly used for industrial purposes to address the worldwide demands for bio-based products (Gunstone 2008; Lee *et al*. 2015). For example, hydroxy FAs (HFAs) are produced in the seeds of a few plant species, with widespread applications in industry such as anticorrosion coatings, nylon, plastics, paints, cosmetics, soaps, detergents, and fuel additives (Binder *et al*. 1962; Lee *et al*. 2015; Ohlrogge *et al*. 2018). The formation of HFA estolides via esterification with other FAs enhances their commercial value and utility with favorable cold flow properties at low temperatures (Cermak *et al*. 2006). Castor (*Ricinus communis* L.) seed oil contains 80–90% HFAs in the form of ricinoleic acid or C18:1-OH (denoted as number of carbons: number of double bonds) (Cocuron *et al*. 2014). Unfortunately, castor production in the United States is generally banned for commercial use due to the presence of the toxin ricin along with potent allergens capable of triggering asthma and other severe responses (Davison *et al*. 1983; Thorpe *et al*. 1988; Cocuron *et al*. 2014).

*Physaria fendleri* (2*n* = 12) is another HFA-producing plant species and a member of the Brassicaceae family native to the arid southwestern United States and northern Mexico. While its seeds accumulate the HFA lesquerolic acid (C20:1-OH), its genetic similarity to other common crops (e.g. canola and *Camelina sativa*) and model species (e.g. *Arabidopsis thaliana*) has led to increased research as a model for HFA production and potential domestic replacement for imported castor oil (Horn *et al*. 2016; Mandáková *et al*. 2017; Azeez *et al*. 2022; Cocuron and Alonso 2023). While castor can produce variable amounts of HFAs, it is generally universally reported to be higher than *P. fendleri*. Previous literature reports Chilean castor accessions produce 46–54% of seed weight as oil, and Indian accessions are reported to range from 38 to 55% seed oil, with a maximum of 90% of total seed FAs comprised C18:1-OH (Anjani 2012; Román-Figueroa *et al*. 2020). In contrast, *P. fendleri* generally only produces about 25% of its seed weight as oil, with a maximum of 60% FA comprising C20:1-OH (Barclay *et al*. 1962; Cocuron *et al*. 2014). Due to these limitations, metabolic engineering and breeding are necessary for the full introduction of HFAs derived from *P. fendleri* into industry (Salywon *et al*. 2005; Azeez *et al*. 2022). Such efforts must aim to improve total oil quantity and/or C20:1-OH content as suitable for the aforementioned industrial applications.

Although the genome of *P. fendleri* has been partially constructed to study interspecies variation, a genome of high enough contiguity and precision for structural and thus functional annotation is lacking (Kiefer *et al*. 2019). The high frequency of outcrossing of this species results in additional challenges due to great sequence variation among individuals, especially when using integrated evidence. With advances in the integration of various sequencing read types, reference-directed genome assembly methods, and strategies for managing intraspecies/intrasample sequence variation, a collection of diverse data types can be effectively used for constructing a high-confidence genome assembly based on established workflows (Roach *et al*. 2018; Sohn and Nam 2018). These workflows include scaffolding and gap-filling tools that can not only make use of paired or long DNA reads but also RNA-seq reads of varying lengths (Xue *et al*. 2013; Song *et al*. 2016). The closing and filling of gaps can also be done using preassembled contigs, which is helpful to obtain higher confidence by using multiple assembler algorithms (Kosugi *et al*. 2015; Xu *et al*. 2020). Newer validated programs are able to correct potential misassemblies and rescaffold based on whole-reference genomes, including those of closely related species; this can then be followed by placing of scaffolds to pseudochromosomes using synteny and homology, producing validated references without optical mapping or Hi-C reads (Tamazian *et al*. 2016; Alonge *et al*. 2022). Finally, in addition to analyzing contiguity and read representation, genomes (and associated transcriptomes) can be carefully evaluated from multiple perspectives including expected ortholog content, completeness of coding regions, and synteny comparisons with related species (Reese *et al*. 2014; Reuscher *et al*. 2018; Manni *et al*. 2021). These processed genome drafts, along with publicly available sequence data of all types, are then effective inputs for gene prediction using canonical approaches employed by the MAKER pipeline, namely Augustus and SNAP (Korf 2004; Stanke *et al*. 2006; Campbell *et al*. 2014b; Hoff and Stanke 2019).

The objective of this work was to assemble a structurally annotated draft reference for *P. fendleri* by combining short paired-end genomic and RNA-seq reads, long RNA-seq reads, and preassembled contigs, including experimental and publicly available data. The functional annotation of this reference data set should provide oilseed researchers with a new tool for analyzing HFA metabolism and identifying potential targets for metabolic engineering. Finally, the strategy used for the construction of this draft aims to address some of the challenges of working with outcrossing plant species, such as the erroneous creation of duplicated regions and the inability to resolve haplotypes (Dierig *et al*. 2012; Roach *et al*. 2018).

## Materials and methods

### Plant materials and nucleic acid extraction

*P. fendleri* embryos were cultured in vitro in accordance with Cocuron and Alonso (2023), with minor modifications, to reduce variance in environmentally induced gene expression and normalize RNA yield. Embryos used for culturing were collected from mother plants of the WCL-YS1 (USDA accession: PI 610492) cultivar at 21 days after pollination, with embryos being excised from the seed coat prior to use (Dierig *et al*. 2000). Mother plants were grown from seed in a growth chamber set to 22°C, 320 µE m^−2^ s^−1^, and 50% relative humidity with a 16-h photoperiod. Culture medium consisted of 40 mM glucose, 5 mM glutamine, 2.0 µl ml^−1^ of 1,000× Gamborg vitamin solution, 20 mM HEPES, 2.2 mg ml^−1^ of Murashige & Skoog basal salts, 0.02 mM of (+/−) abscisic acid, and 23% (*w*/*v*) polyethylene glycol. This media resulted in a production of FA quantity and composition similar to that of the in planta embryos, approximately 25% *w*/*w* of total oil of which ∼55% was lesquerolic acid, when cultured for 9 days (Supplementary Tables 1 and 2). Cultures were maintained in an incubator set to 12 µE m^−2^ s^−1^ at 22°C and 50% relative humidity.

RNA was extracted from cultured *P. fendleri* embryos using a 2.0% *w*/*v* hexadecyl(trimethyl)azanium bromide (CTAB) extraction buffer containing a 2 M concentration of sodium chloride, 100 mM of 2-amino-2-(hydroxymethyl)propane-1,3-diol dihydrochloride (Tris-HCl) at pH = 8, 25 mM of 2-[2-[bis(carboxymethyl)amino]ethyl-(carboxymethyl)amino]acetic acid (EDTA) at pH = 8, and 3.0% *w*/*v* 1-ethenylpyrrolidin-2-one (PVPP) according to Johnston *et al*. (2022). After adding 2-sulfanylethan-1-ol and N1-(3-aminopropyl)butane-1,4-diamine (spermidine) to the buffer in order to achieve a concentration of 3.0% *v*/*v* and 0.5 g L^−1^, respectively, the buffer was heated to 65°C prior to extraction. Two sequential extractions were performed with 600 and 450 µL per sample. Following each extraction, a 24:1 (*v*/*v*) trichloromethane:3-methylbutan-1-ol solution was added and aqueous layers were combined in tubes on ice following centrifugation. This aqueous phase was further subject to phase separation with 1 equal volume of the 24:1 (*v*/*v*) trichloromethane:3-methylbutan-1-ol solution, with aqueous phase being collected in tubes on ice. Ethanol precipitation was carried out on the final aqueous extract by adding 0.10 volumes of sodium acetate, 0.25 volumes of RNA-grade glycogen (0.5 mg mL^−1^), and 2 volumes of ethanol prior to incubation at −20°C for 12 h. Following precipitation, extracts were centrifuged for 1 h at 13,000 × *g* and 4°C and RNA pellets were rinsed 3 times with 1 ml of 70% (*v*/*v*) ethanol and dried under a sterile bench. Samples were resuspended in RNase-free water and 0.3 volumes of 8 M lithium chloride were added prior to incubation at −20°C for 12 h. An additional 3 rinses with 70% (*v*/*v*) ethanol were carried out after centrifuging for 1 h at 13,000 × *g*, following which RNA pellets were resuspended in RNase-free water.

### Library preparation and evidence sources

**Fig. 1.**
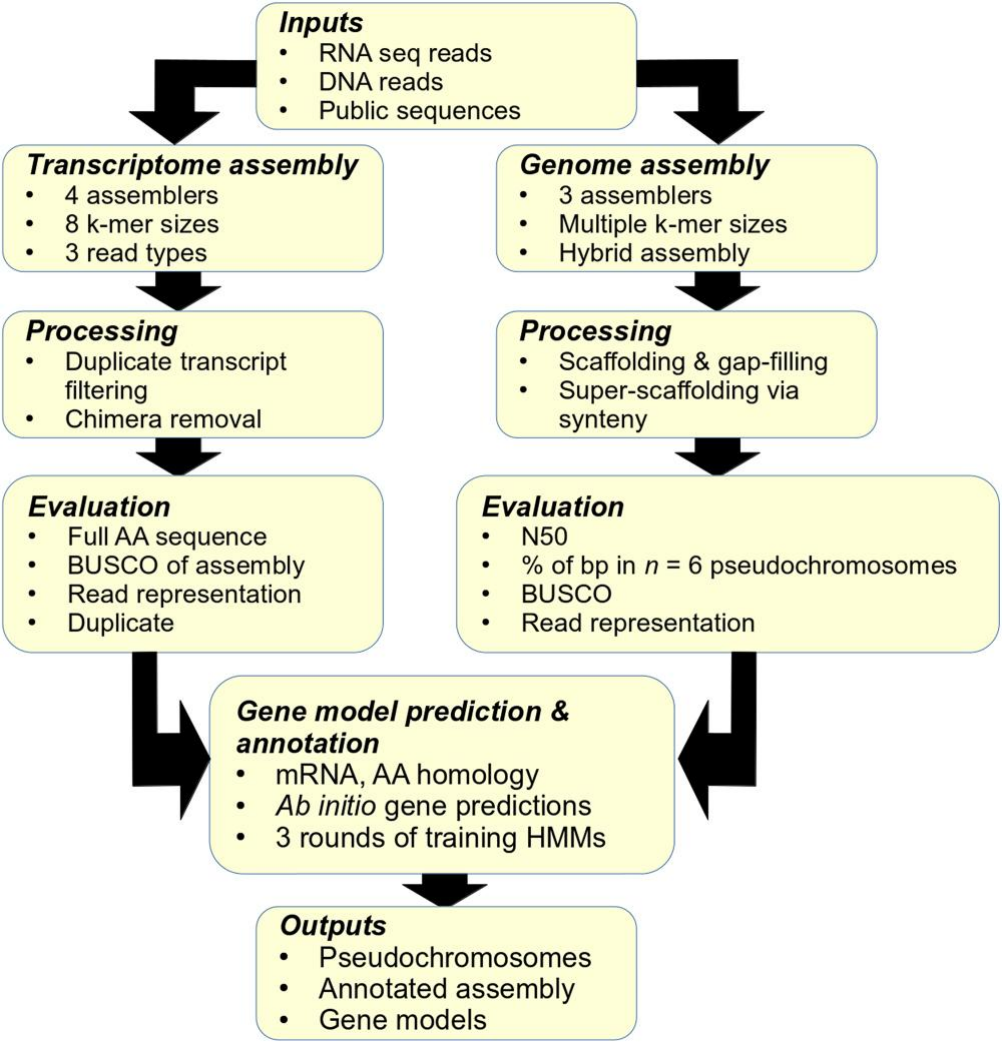
Summary of genome and transcriptome assembly workflow. Specifics about each step can be found in Supplementary Figs. 2 and 3.

An integrated methodology using both generated RNA-seq reads and available genomic and RNA-seq reads from public databases (described below) was undertaken to produce a stronger consensus assembly (Fig. 1). For generated RNA-seq data, extracted samples were analyzed using capillary electrophoresis to determine RNA integrity and detect any contamination (Supplementary Fig. 1). Six replicates were used with approximately 500 ng of RNA for each replicate. The total library of 2 × 75-bp reads was prepared using the Illumina Stranded Total RNA Prep workflow and the NextSeq MidOutput (Illumina, San Diego, CA, USA) kit. Paired-end reads were generated and subjected to FastQC for quality control prior to usage (Sequencing Read Archive [SRA]: SRP430430). Additional *P. fendleri* read data from the NCBI SRA (https://www.ncbi.nlm.nih.gov/sra) was used for both genomic and transcriptomic assemblies. For the transcriptome assembly, RNA-seq data from single-end Illumina reads (BioProject: PRJNA291630; SRA: SRP061893) and 454 long reads (BioProject: PRJNA260225; SRA: SRP046070) were also integrated into the assembly process as described below (Kim and Chen 2015; Horn *et al*. 2016). For the genomic assembly, paired-end Illumina reads (BioProject: PRJEB26555; SRA: ERP108550) and the associated fragmented genome assembly of *P. fendleri* (GenBank Acc.: GCA_900406525.1) were used as described below (Kiefer *et al*. 2019). These genomic reads were run in the MaSuRCA assembler (v4.1.0) with *k*-mer coverage ranging from *k* = 49 to *k* = 75 to produce super-reads for additional contig evidence (Zimin *et al*. 2013).

### De novo transcriptome assembly

A de novo transcriptome assembly was prepared by utilizing multiple assembler programs, *k*-mer sizes, data sets, and filtering algorithms (Supplementary Fig. 2). The effect of *k*-mer size (Supplementary Fig. 3) was first observed using SOAPdenovo-Trans at *k* = 25, 27, 29, 31, 33, 35, 37, and 39 with the aforementioned paired-end library (Xie *et al*. 2014). To ensure the inclusion of assembled transcripts detectable with a certain *k*-mer size, all 8 *k*-mer sizes were merged using the “transabyss-merge” function in Trans-ABySS (v2.0.1) (Robertson *et al*. 2010). Additional assemblies were performed on paired-end read data using rnaSPADES (“--ss rf” for strand-specific reads), Trans-ABySS (default settings), and Trinity (“--SS_lib_type RF”) (Robertson *et al* 2010; Grabherr *et al*. 2011; Bushmanova *et al*. 2019). To improve assembly quality, the public RNA-seq data (the single-end Illumina reads and the 454 long reads) were also analyzed separately through each assembler and merged, tuning parameters as necessary to account for the different read types.

Output from all 4 assemblers and all 3 data sets was merged using transabyss-merge, followed by an initial removal of duplicates via the “tr2aacds” function from EvidentialGene (http://arthropods.eugenes.org/EvidentialGene/) and one pass through CD-HIT-EST at an identity cutoff of 0.98 (Li and Godzik 2006). The resulting draft assembly was then passed through a modified version of the Bellerophon pipeline, in which reads were mapped to the draft with HISAT2 and transcripts comprising a sum of under 1 transcript-per-million across all samples were removed, followed by another run of CD-HIT-EST with an identity cutoff of 0.95 (Huber *et al*. 2004; Kim *et al*. 2015). Finally, chimera removal was carried out using the method developed by Yang and Smith (2013). For transcriptome assembly evaluation, the “brassicales_odb10” database was used since it was considered most precise for protein-coding gene evaluation in a Brassicaceae (Manni *et al*. 2021). All associated ORFs were detected using TransDecoder (v5.5.0) (https://github.com/TransDecoder/TransDecoder), integrating homology detected via UniProt and Pfam matches in order to produce predicted amino acid sequences (Bateman *et al*. 2023; Mistry *et al.* 2021).

### Organellular genome assembly

Organellular genomes were constructed from the aforementioned genomic reads (Supplementary Fig. 4). The chloroplast genome was assembled at *k* = 85 using GetOrganelle (Jin *et al*. 2020). The mitochondrial genome was assembled using SPADES with *k* = 55 after testing from *k* = 33 to *k* = 95 (Supplementary Table 3) (Bankevich *et al*. 2012). The initial *k* = 55 assembly was first scaffolded using the genomic super-reads from MaSuRCA in LRSCAF and then was scaffolded around exonic regions by using RNA-seq data, with L_RNA_scaffolder (Xue *et al*. 2013; Qin *et al*. 2019) and P_RNA_scaffolder for the public long-read RNA-seq data and generated paired-end Illumina RNA-seq data, respectively (Zhu *et al*. 2018). Further scaffolding was carried out within protein-coding regions via PEP_scaffolder, using the predicted amino acid sequences from the de novo transcriptome (Zhu *et al*. 2016). A second de novo assembly was performed using GetOrganelle with *k* = 85 (Xu *et al*. 2020). This second de novo mitochondrion assembly was merged with the scaffolded SPADES assembly via MAC.

### Nuclear genome assembly

The existing fragmented assembly was then used as an initial foundation for nuclear genome construction (Supplementary Fig. 4). The associated genomic reads were initially used to create secondary assemblies using 3 different assemblers: ABySS at *k* = 66 and *k* = 72, SOAPdenovo (r242) at *k* = 63 and *k* = 71, and MaSuRCA with auto-k (converged on *k* = 67) at *k* = 49 and *k* = 75 (Supplementary Table 4) (Luo *et al*. 2015; Jackman *et al*. 2017). All secondary assemblies along with the fragmented genome assembly were merged with MAC and then subjected to 3 rounds of scaffolding and gap filling with both genomic and transcriptomic data, each round being separated by a run in Purge Haplotigs to deal with the high heterozygosity (Supplementary Table 1) (Roach *et al*. 2018).

Following scaffolding and gap filling, the resulting 24,235 contigs were placed into pseudochromosomes according to synteny with the 6 chromosomes (same *n* as *P. fendleri*) of the *Camelina laxa* assembly (GenBank Acc.: GCA_024034495.1) using the Chromosemble tool from Satsuma (Grabherr *et al*. 2010; more information in Supplementary Table 3). The *C. laxa* target assembly was first masked according to the following. Miniature inverted-repeat transposable elements (MITEs) were detected using MITE-Tracker and combined with long terminal repeats (LTRs) detected according to Campbell *et al*. 2014b). More evolutionarily recent LTR retrotransposons were detected at a similarity 99% and were combined with older LTRs detected with a reduced similarity of 85%. Insertions nested within LTR elements were identified and likely false-positive elements in internal LTR regions were masked. The resulting LTR and MITE libraries were combined with repeats identified by RepeatModeler, and subsequently run against a database of known *A. thaliana* proteins (GenBank Acc.: GCA_000001735.2) manually cleared of transposable elements using BLASTx, and these BLAST hits were excluded from the repeat library (Camacho *et al*. 2009; Flynn *et al*. 2020). The combined repeat library was then used for masking the target *C. laxa* assembly prior to contig placement. The resulting assembly was subjected to 1 last round of scaffolding and gap filling, primarily to join unplaced contigs, resulting in 90.4% of the final 272,649,109-bp assembly residing on the first 6 pseudochromosomes.

### Annotation

A limited functional annotation was carried out on the de novo transcriptome assembly using the Trinotate pipeline, following which only final sequences were kept for later usage by the MAKER pipeline (Campbell *et al*. 2014a, 2014b; Bryant *et al*. 2017). Prior to annotation, the nuclear genome was first scanned for sequence repeats according to the same methodology described above for super-scaffolding. Following this, the genome assembly quality was also evaluated by the LTR assembly index as described by Mokhtar *et al*. (2023). Gene prediction and annotation of the nuclear genome were carried out via 3 rounds of the MAKER pipeline. For the first round, the de novo transcriptome assembly and associated protein predictions were used as mRNA and protein homology evidence, respectively, and the combined repeat library was used for masking (Supplementary Table 5). Specifically, homology was detected by using BLAST hits against the reference proteomes (via BLASTp) of *A. thaliana* (Refseq Acc.: GCF_000001735.4), *C. sativa* (RefSeq Acc.: GCF_000633955.1), *Brassica rapa* (RefSeq Acc.: GCF_000309985.2), *Raphanus sativus* (RefSeq Acc.: GCF_000801105.2), and *Brassica napus* (RefSeq Acc.: GCF_020379485.1). A reference-free run of rnaQUAST was used with GeneMarkS-T to estimate the number of genes giving rise to the de novo transcripts assembled (Tang *et al*. 2015; Bushmanova *et al*. 2016).

In the first round, ab initio predictions were directly inferred from the provided mRNA and protein sequence information, following which predictions were exported to SNAP and Augustus for training hidden Markov models (HMMs) (Korf 2004; Stanke *et al*. 2006; Hoff and Stanke 2019). The HMMs produced by SNAP and Augustus were fed back into a second round of MAKER for refining gene predictions, on which SNAP and Augustus were retrained for a second iteration, finally producing input predictions for the third round of MAKER. The chloroplast genome and mitochondrial genome were annotated using GeSeq and Mitofy, respectively (Alverson *et al*. 2010; Tillich *et al*. 2017).

### Synteny analysis

Analysis of interspecies genomic synteny within the *P. fendleri* assembly was carried out on both *A. thaliana* and *C. laxa* (GenBank Acc.: GCA_024034495.1) genomes using the Satsuma2 package (https://github.com/bioinfologics/satsuma2) with a minimum block size of 10 (Berardini *et al*. 2015). MCScanX was also used to examine collinearity within the constructed *P. fendleri* genome based on amino acid sequence alignments with BLASTP at *E*-value threshold of 1 × 10^−5^ (Wang *et al*. 2012). For interspecies synteny, syntenic blocks contained a minimum identity of 85.0%, a minimum length of 2009 bp (the average transcript length from the MAKER output), and a maximum gap size of 1 Mb for joining blocks on the same strand. For collinearity within *P. fendleri*, syntenic blocks had a minimum length of 1 Mb, and blocks were joined on the same strand within pseudochromosomes using a maximum gap size of 1 Mb. Syntenic blocks were not considered if the query and target sequence length differed by more than 3-fold.

## Results and discussion

### Transcriptome assembly performance

A total of 386,008,498 paired-end reads were generated for the transcriptome assembly. As no contiguous *P. fendleri* assembly of reference quality was available, only de novo transcriptome assembly was performed. To converge on a consensus assembly, a factorial combination of 4 assemblers and 3 different types of reads were used and evaluated by read representation (defined as the overall alignment rate of all reads used against the final assembly), the total number of transcripts, and a rough estimate of the number of genes predicted to give rise to those transcripts. Upon evaluation of the impact of *k*-mer size, all combinations of RNA-seq reads tested clearly displayed a preference for *k* = 31. However, this was only evident when paired-reads were analyzed alone and when evaluated by the gross number of total sequences yielded (Supplementary Fig. 2). The addition of new read types buffered the effect of *k*-mer size; however, based on the results, it can be seen that overall, read type as well as the assembler used had a much large effect on the number of sequences assembled and the mean sequence length. Since a robust filtering procedure was used later on and the effect of *k*-mer size was modest, all tested *k*-mer sizes were merged via transabyss-merge to form the representative SOAPdenovo-Trans assembly.

**Fig. 2.**
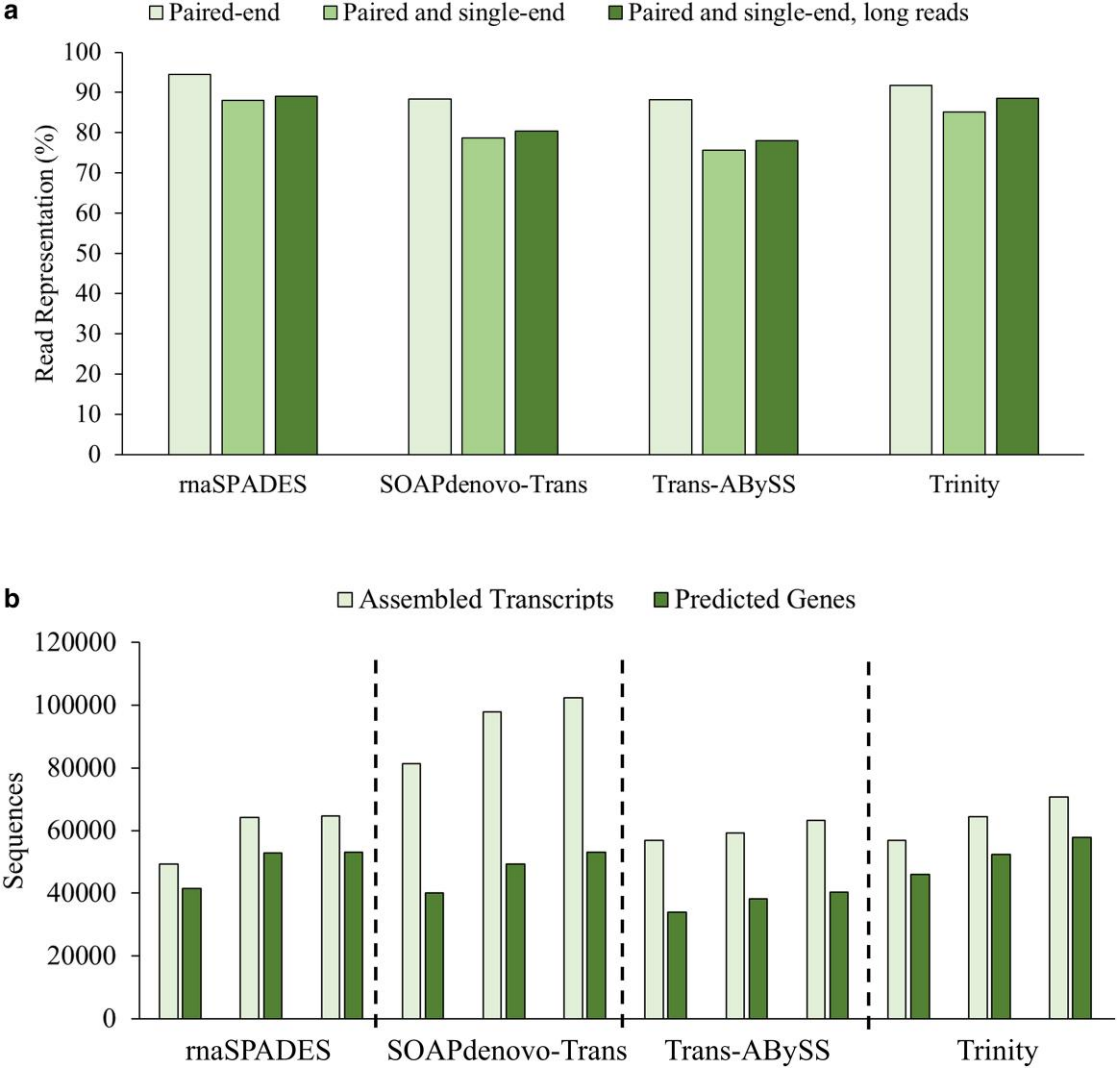
Organellular genome annotations of *P. fendleri*. a) Chloroplast genome (Genbank Acc.: BK063463). b) Mitochondrial genome (GenBank Acc.: OQ818196).

Although all assemblers were able to produce assemblies with >88.0% read representation when using only paired-end reads, the magnitude of the effect of adding new read types differed across assemblers despite a consistent trend within each assembler (Fig. 2). Trinity handled sequential addition of other read types the most effectively, only losing 7.0% read representation when adding single-end Illumina reads, and recovered to a total loss of only 3.5% read representation when long RNA-seq reads were added to both. Trans-ABySS produced the assemblies with the lowest read representation regardless of read type. It was found that merging the assembly created with all 3 read types, from each assembler, produced a set of 199,563 transcripts (of which only 68.0% were over 500 bp), highlighting the necessity for further processing (Supplementary Table 6). While 86.6% of expected BUSCOs were found complete, 75.9% were duplicated, which is likely high for a diploid Brassicaceae considering the reference-quality *A. thaliana* TAIR10.1 assembly contains 43.1% complete BUSCOs as duplicates (Berardini *et al*. 2015). Regardless, a high read representation of 93.6% was obtained from the merged, unfiltered assembly.

To remove bulk fragments/artifacts and duplicated transcripts, the prefilter chosen, tr2aacds followed by CD-HIT-EST, reduced the total transcript count to 70,840 and the number of duplicated complete BUSCOs to 58.1% (Supplementary Table 7). A total of 73.9% of the BLASTX hits of the prefiltered assembly to TAIR10.1 protein products were full length (>80.0% query cover). The 3-step final processing procedure was then performed for a more precise removal of duplicates, fabricated transcript isoforms, and chimeras that was not subject to overpowering by the noise of bulk fragments and artifacts removed by prefiltering. Following all 3 steps of final processing, a final de novo transcriptome was yielded containing 74,416 total transcripts, 91.4% read representation, with 77.9% of BLASTX hits considered complete (>80.0% query cover), and 87.8% of expected BUSCOs complete, with 62% duplicated. The mean sequence length of the final assembly was 1,661 bp, comparable to that of the transcriptome of *A. thaliana* (1,663 bp) (Berardini *et al*. 2015). Following annotation with the Trinotate pipeline, the assembly increased to 75,096 transcripts, assumed to be additional splice variants detected by the TransDecoder step and homology. The transcriptome assembly was considered an independent piece of evidence for arriving at the draft *P. fendleri* genome, having been performed de novo.

### Chloroplast and mitochondrial genomes

**Fig. 3.**
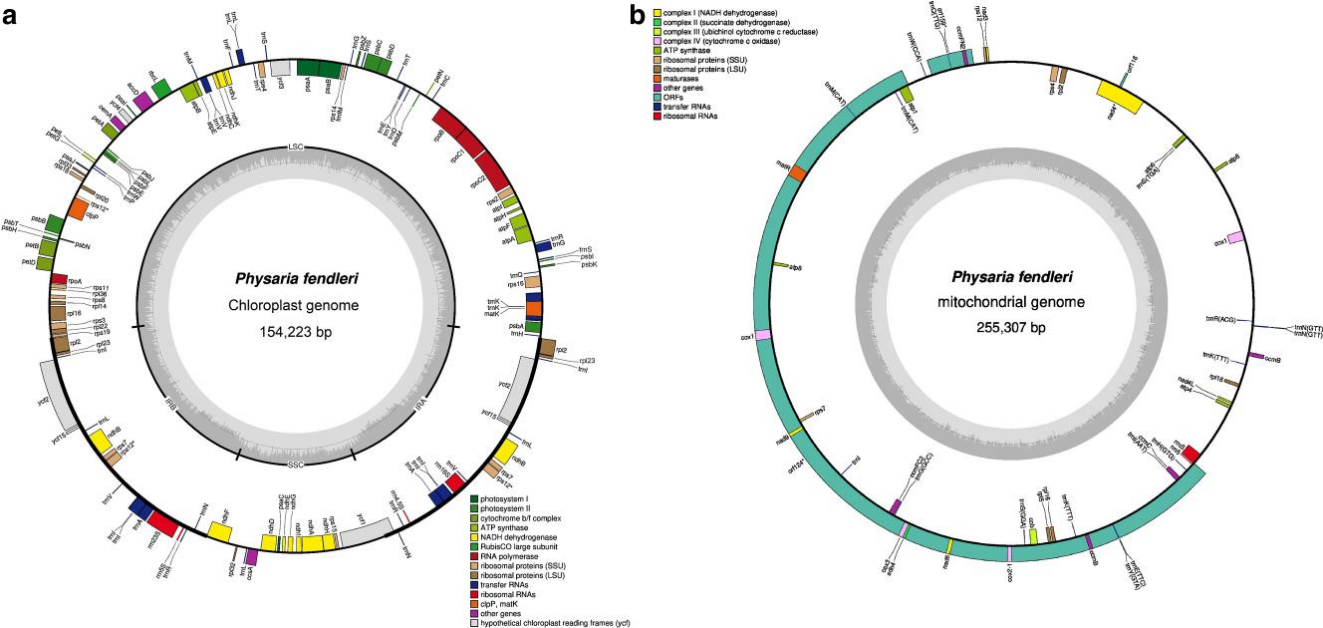
De novo transcriptome assembly evaluation. a) Effect of assembler and read combinations on read representation, defined as the overall alignment rate of generated reads that mapped back to the produced assembly. Paired-end = SRA: SRP430430; single-end = SRA: SRP061893; long reads = SRA: SRP046070. b) Effect of assembler on total transcripts produced, and total corresponding genes giving rise to the transcripts as predicted by GeneMarkS-T.

The chloroplast genome (Genbank Acc.: BK063463) was assembled with 391× coverage, and produced a single closed contig without unplaced scaffolds (Fig. 3). The accuracy of the chloroplast assembly was validated by >99.7% identity with the chloroplast of *Physaria pinetorum* (GenBank Acc.: MK637778.1). The mitochondrial genome (GenBank Acc.: OQ818196) was assembled with 262× coverage; however, the SPADES assembly alone did not produce a useable contiguous sequence even with the optimal *k*-mer size and further scaffolding (35 contigs in best assembly). Merging the additional GetOrganelle assembly with the SPADES one using MAC, 8 contigs were achieved; one last run of LRSCAF, L_RNA_scaffolder, and P_RNA_scaffolder as previously described produced a final contig of 255,307 bp (Fig. 3) (Tang *et al*. 2020).

### Step-wise genome assembly performance

The SRA genomic reads were reassembled and merged using a similar strategy to that of the transcriptome assembly; multiple assemblers were used; however, for each, 2–3 *k*-mer sizes were used. While ABySS had the best performance in terms of N50, it still only had a maximum N50 of 2,783 at *k* = 66. The same assembly had the lowest contig count (*n*) of all, but it was still highly fragmented with 140,873 contigs. Due to this fragmented pattern of assembly output, an integrated approach was taken to deal with scaffolding and eventual gap filling. Merging all de novo assemblies into a consensus assembly via MAC reduced the total *n* to 103,241 and achieved an N50 of 6,982. A first round of scaffolding and gap filling yielded an assembly with N50 of 9,871 and *n* of 89,502; in comparison, the fragmented *P. fendleri* assembly had only ∼10.0% more contigs, indicating an issue with the de novo assemblies.

**Fig. 4.**
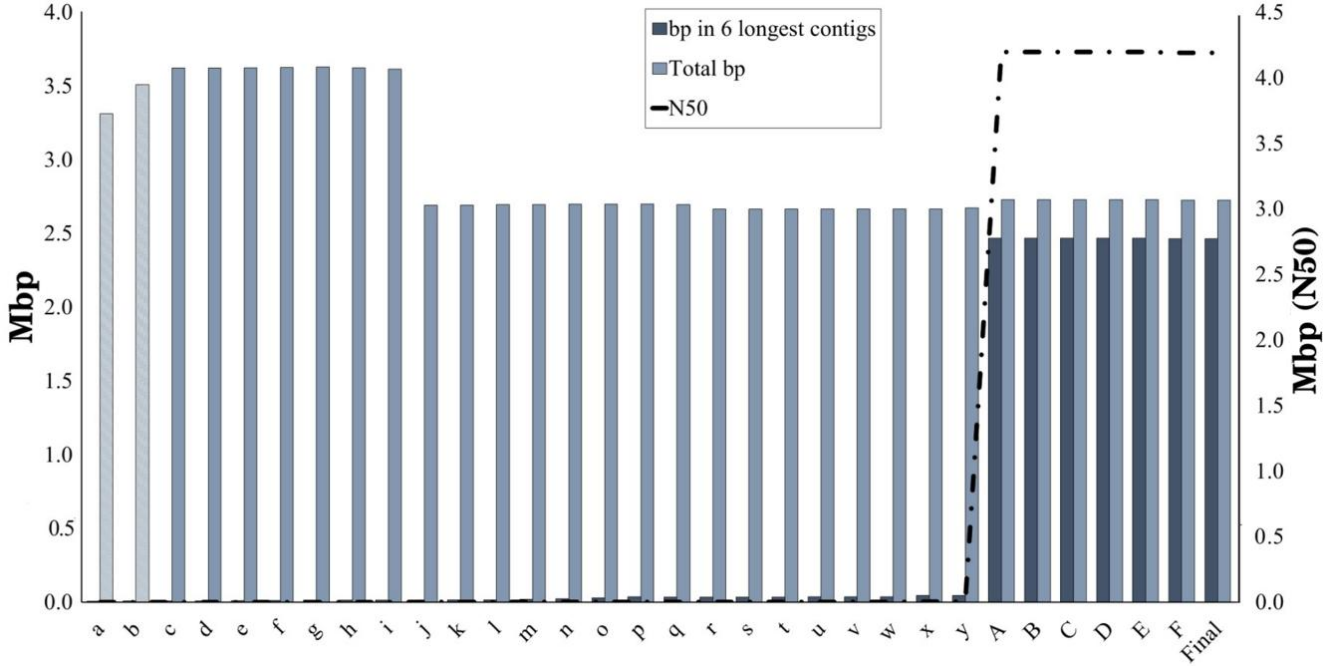
Quantitative evaluation of genome size at each processing step in assembly workflow. The left axis corresponds to the base pair in the 6 longest contigs, as well as the total base pair in the assembly including unplaced scaffolds. The right axis corresponds to the N50 at each step. See Supplementary Table 3 for details on each step.

As *P. fendleri* is an obligate outcrosser, it was expected to have a significant amount of haplotigs, likely inflating the true size of the genome (Dierig *et al*. 2012; Olson *et al*. 2015). This was determined to likely be the case, as the estimated genome size using the *k*-mer distribution with ntcard (Supplementary Fig. 5) indicated a mean *k*-mer coverage of 18.8, heterozygosity of 2.4%, and an estimated total genome length of 324 Mb (*k* = 26) (Mohamadi *et al*. 2017). Similarly, the fragmented assembly available online was 331 Mb. As a result of suspected haplotig content, the assembly was subjected to a run of Purge Haplotigs at a low read depth cutoff of 2, mid-point of 29, and high cutoff of 190, with a default 70.0% cutoff for assigning a contig as a haplotig. Remarkably, the *n* of the assembly dropped to 34,718, with an N50 of 14,432, and the total size of the genome was reduced to 269 Mb (Fig. 4). The final 2 rounds of gap filling, split by another precautionary run of Purge Haplotigs (low cutoff of 2, mid-point of 25, and high cutoff of 172 in accordance with the read depth histogram), was carried out. Only modest improvements were observed in terms of total contig count, N50, and gaps with each successive step in the final 2 rounds; however, following completion, a scaffold-level assembly with *n* of 24,235, N50 of 33,600, and 267 Mb in size was produced. The N90 at this level of the assembly was 4,563 bp, while the average transcript length in *A. thaliana* is 1,663 bp; due to this contiguity in gene-coding regions (BUSCO “embryophyta_odb10”: 77.3% complete) and the low count of total bases in gaps throughout the assembly (1.7%), contigs were subsequently super-scaffolded (Berardini *et al*. 2015). Concerning read representation, 84.0% of the paired-end RNA-seq reads and 78.2% of the genomic reads from the NCBI SRA mapped back to the final assembly (Table 1). This slight drop in representation of genomic reads is likely a result of haplotig content.

**Table 1.** Final assembly results, including 6 pseudochromosomes, mitochondrial assembly, and chloroplast assembly.

Assembly statistics	N50	41,908,749
	Total bp	246,881,883
	Total contigs	8
	RNA-seq read rep.*^a^*	83.95%
	DNA read rep.*^b^*	78.19%
	Gaps	87248
	% *N*	3.92
Annotation results*^c^*	Gene models	39,859
	Avg. gene length	2,009.39
	% genes AED < 0.5	0.873
	Complete BUSCO hits	77.10%

^*a*^RNA-seq read representation, with reads mapped via bowtie2 “--local”. SRA: SRP430430.

^*b*^DNA read representation, with reads mapped via bowtie2 “--local”. SRA: ERP108550.

^*c*^Annotation results correspond only to 6 pseudochromosomes, chloroplast, and mitochondrial genomes.

**Fig. 5.**
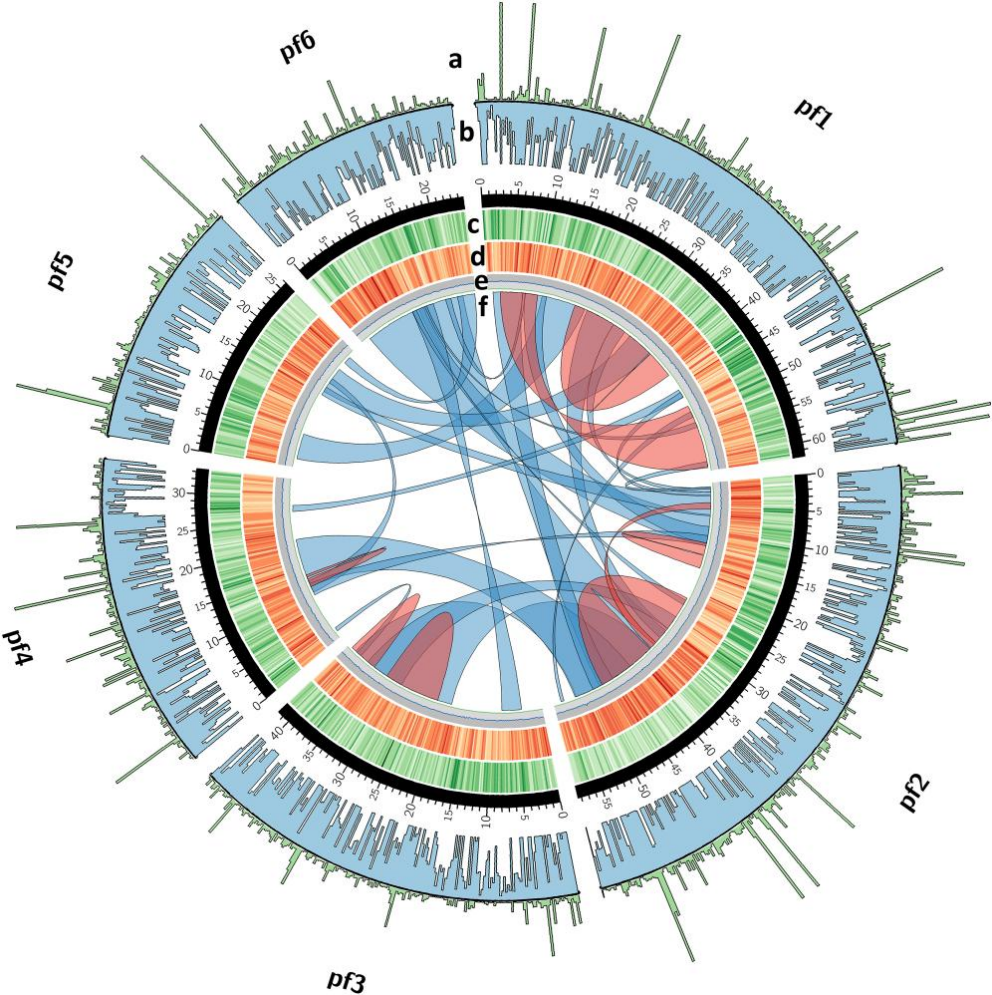
Genomic topography of the 6 pseudochromosomes of *P. fendleri*. Plot created using Circos (Krzywinski *et al*. 2009). a) Read depth of paired-end RNA-seq reads (SRA: SRP430430) in 200,000-bp intervals, maximum of 500 bp shown. b) Coverage of genomic paired-end reads (SRA: ERP108550) in 200,000-bp intervals, maximum of 40× shown. c) Repeat density, represented as the percentage of bases in identified repeats for each 200,000-bp interval. d) Gene density, represented as the number of identified protein-coding genes per 200,000 bp. e) GC content for each 200,000-bp interval. The inside line next to f) represents 0%, and the maximum is 66%. f) Inter- and intrachromosomal synteny detected via MCScanX (Wang *et al*. 2012). Minimum block size of 1 Mb, with each 1 Mb containing a majority of base pair in a syntenic block considered for linking.

The final assembly contained a BUSCO completeness of 77.1% when including the chloroplast and mitochondrial genomes, but removing unplaced scaffolds (Table 1); this being so close in comparison to the BUSCO completeness of the fragments prior to super-scaffolding (*n* = 24,235) confirms that placement into the masked *C. laxa* genome did not distort or lose significant protein-coding gene structure (Table 2). Of the genes annotated by MAKER, 87.3% had an Annotation Edit Distance of under 0.5, comparable to well-annotated de novo genomes including those of diploid plants (Eilbeck *et al*. 2009; Campbell *et al*. 2014a; Sork *et al*. 2016). Gene density was shown to be generally well correlated with RNA-seq read representation on a global scale (Fig. 5).

**Table 2.** Results of individual contigs, not including unplaced scaffolds.

Contigs				
Contig	bp	Gaps	*N*s	% *N*
1	61,969,647	22,538	2,406,911	3.88
2	58,466,665	19,556	2,017,778	3.45
3	41,908,749	15,456	1,626,681	3.88
4	33,349,723	11,535	1,358,494	4.07
5	26,039,851	9429	1,210,825	4.65
6	24,737,718	8728	1,058,287	4.28
Cp	154,223	0	0	0.00
Mt	255,307	6	600	0.24

### Integrity of draft assembly strategy

**Fig. 6.**
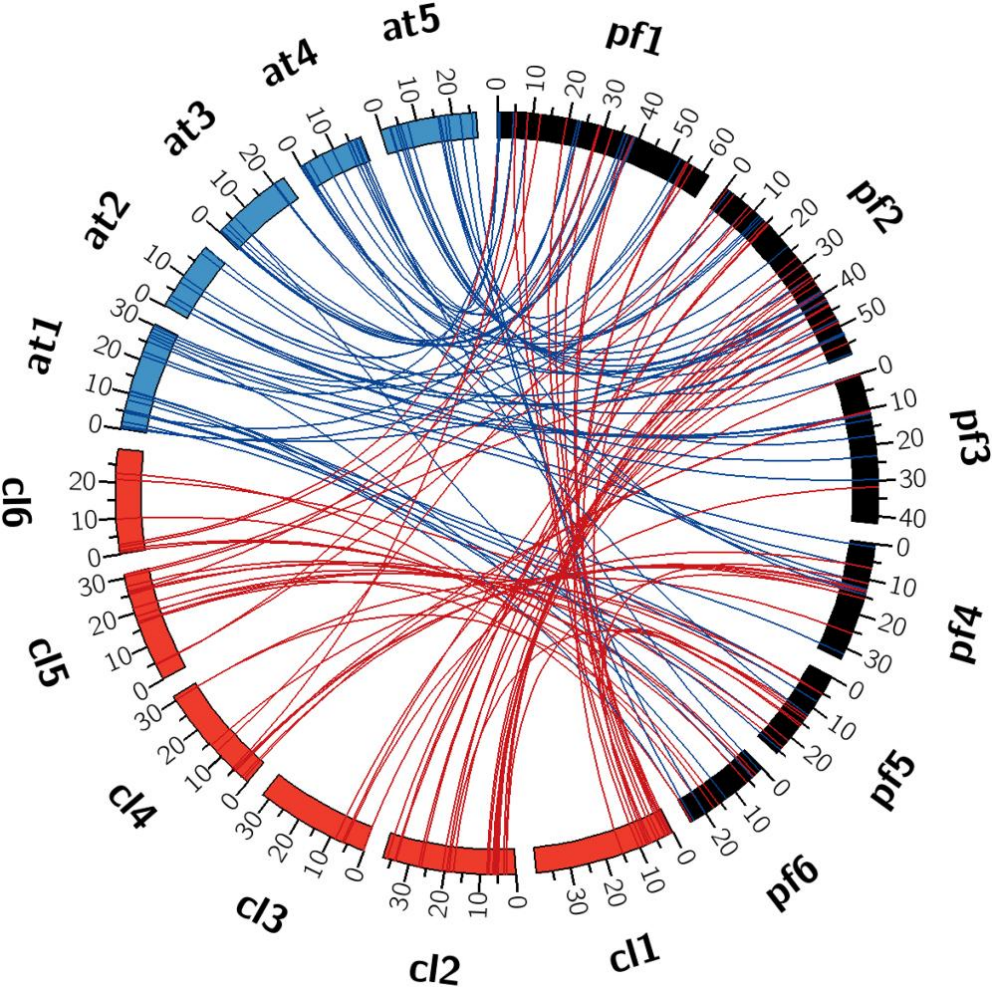
Interspecies synteny between *P. fendleri* and *C. laxa* (GenBank Acc.: GCA_024034495.1) and *A. thaliana* (Berardini *et al*. 2015). Detected via Satsuma2 (https://github.com/bioinfologics/satsuma2). Red links indicate synteny between *P. fendleri* and *C. laxa*, blue links indicate synteny between *P. fendleri* and *A. thaliana*. Scale of chromosome size is megabase pair (between layers b and c).

The topography of the genome assembly with respect to the gene density and repeat density illustrates this strategy was likely effective in preserving a significant degree of the genome structure of *P. fendleri* (Fig. 5). Furthermore, the sanity check that was performed by determining synteny between the unmasked target *C. laxa* assembly and the final *P. fendleri* assembly revealed that genome structure was not simply copied during super-scaffolding; indeed, for each *P. fendleri* pseudochromosome, synteny was observed with multiple *C. laxa* chromosomes (Fig. 6; Supplementary Table 8). Interestingly, synteny between this *P. fendleri* consensus with both *A. thaliana* and *C. laxa* revealed the possible presence of an inversion in several large regions. It is known that Brassicaceae underwent such processes regularly during evolution, particularly the compartmentalization of genomic rearrangements, which was observed in our draft consensus by several large regions of rearrangement when comparing synteny with *A. thaliana* (Murat *et al*. 2015). This trend is especially evident in diploid Brassicaceae, which often contain such compartmented genomic rearrangements after returning to a diploid state following an ancestral polyploidization (Cheng *et al*. 2012, 2014; Murat *et al*. 2015). Regardless, the characterization of this and other evolutionary phenomena would require further analyses for confirming the chromosomal localization of genomic features within the species, particularly optical mapping. The widescale outcrossing of *P. fendleri* allows it to serve as a rich model for studying structural genomics in Brassicaceae, as well as macro- and micro-scale feature comparisons between species. Furthermore, the characterization of *P. fendleri* grants a valuable perspective into the genomic characteristics that contribute to the potential of alternative oilseed crops.

## Supplementary Material

jkae114_Supplementary_Data

## Data Availability

Corresponding data are available under NCBI BioProject PRJNA951248. The *P. fendleri* genome assembly was deposited in GenBank (NCBI Acc.: DAVZGE000000000). Generated paired-end RNA-seq data were deposited in the SRA: SRP430430. Both the chloroplast (Genbank Acc.: BK063463) and mitochondrial genomes (GenBank Acc.: OQ818196) are annotated and available on NCBI. All other third-party SRA data used are publicly available under the accessions listed in the text. Relevant program parameters and code are available in Supplementary Table 9. Supplemental material available at G3 online.
