# Supplementary material for: First draft reference genome and annotation of the alternative oil species Physaria fendleri
Source: G3 (Bethesda). 2024 May 28;14(9):jkae114. doi: 10.1093/g3journal/jkae114 (PMC11373644; doi:10.1093/g3journal/jkae114)
Supplement: jkae114_Supplementary_Data [file jkae114_supplementary_data.zip › Figure_S1_G3-2024-405031.pdf]

A1: Ladder

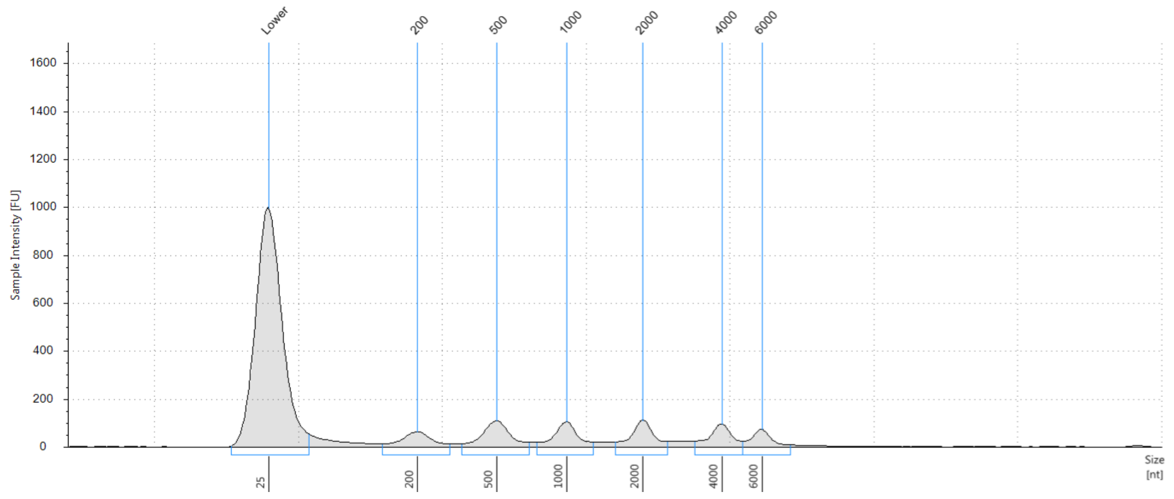

Sample Table

Well	RINe	28S/18S (Area)	Conc. [pg/µl]	Sample Description	Alert	Observations
A1	-	-	307	Ladder	⚠	Caution! Expired ScreenTape device; Markers outside standard running position; Ladder

Peak Table

Size [nt]	Calibrated Conc. [pg/µl]	Assigned Conc. [pg/µl]	Peak Molarity [pmol/l]	% Integrated Area	Peak Comment	Observations
25	700	700	82400	-		edited Lower Marker
200	36.2	-	532	14.27		
500	57.1	-	336	22.50		
1000	46.3	-	136	18.26		
2000	45.9	-	67.5	18.08		
4000	40.5	-	29.7	15.94		
6000	27.8	-	13.6	10.95		

 © Agilent Technologies, Inc. 2016

B1: CJohnston_20220301-1

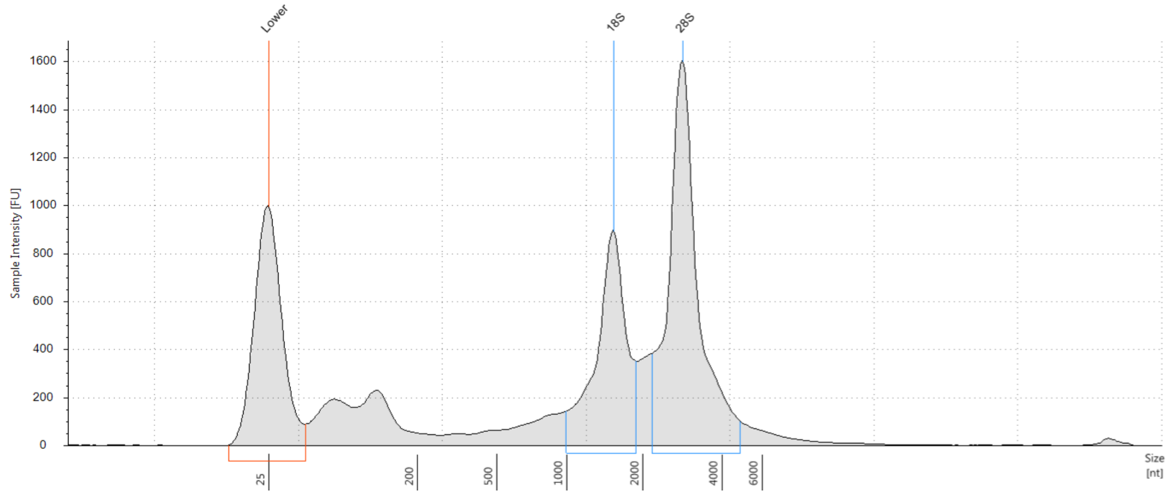

Sample Table

Well	RINe	28S/18S (Area)	Conc. [pg/µl]	Sample Description	Alert	Observations
B1	7.4	1.8	1950	CJohnston_20220301-1	⚠	Caution! Expired ScreenTape device; Markers outside standard running position; RINe edited

Peak Table

Size [nt]	Calibrated Conc. [pg/µl]	Assigned Conc. [pg/µl]	Peak Molarity [pmol/l]	% Integrated Area	Peak Comment	Observations
25	700	700	82400	-		edited Lower Marker
1533	488	-	937	36.09		18S edited
2840	864	-	895	63.91		28S edited

C1: 20220301-2

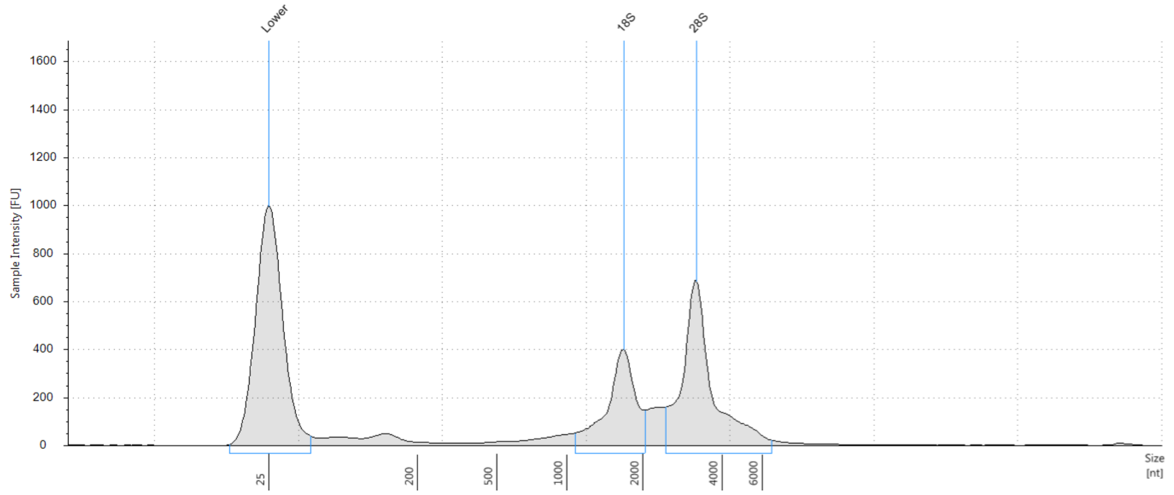

Sample Table

Well	RINe	28S/18S (Area)	Conc. [pg/µl]	Sample Description	Alert	Observations
C1	7.9	1.8	749	20220301-2	⚠	Caution! Expired ScreenTape device; Markers outside standard running position; RNA concentration outside recommended range for RINe; RINe edited

Peak Table

Size [nt]	Calibrated Conc. [pg/µl]	Assigned Conc. [pg/µl]	Peak Molarity [pmol/l]	% Integrated Area	Peak Comment	Observations
25	700	700	82400	-		edited Lower Marker
1687	208	-	363	35.80		18S edited
3204	374	-	343	64.20		28S edited

 © Agilent Technologies, Inc. 2016

D1: 20220301-3

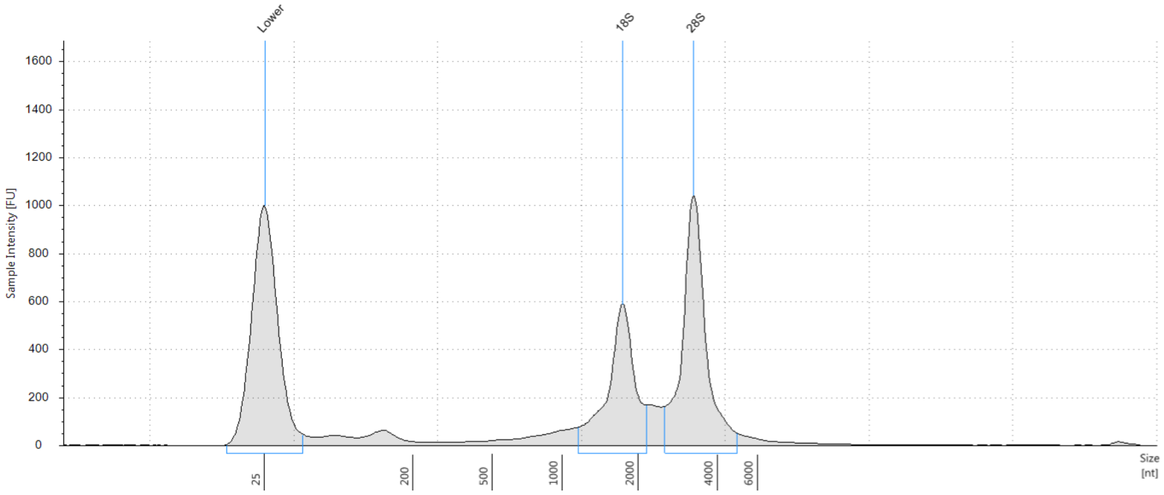

Sample Table

Well	RINe	28S/18S (Area)	Conc. [pg/µl]	Sample Description	Alert	Observations
D1	8.1	1.5	997	20220301-3	⚠	Caution! Expired ScreenTape device; Markers outside standard running position; RNA concentration outside recommended range for RINe; RINe edited

Peak Table

Size [nt]	Calibrated Conc. [pg/µl]	Assigned Conc. [pg/µl]	Peak Molarity [pmol/l]	% Integrated Area	Peak Comment	Observations
25	700	700	82400	-		edited Lower Marker
1730	300	-	511	39.22		18S edited
3244	466	-	422	60.78		28S edited

 © Agilent Technologies, Inc. 2016
