# Supplementary material for: First draft reference genome and annotation of the alternative oil species Physaria fendleri
Source: G3 (Bethesda). 2024 May 28;14(9):jkae114. doi: 10.1093/g3journal/jkae114 (PMC11373644; doi:10.1093/g3journal/jkae114)
Supplement: jkae114_Supplementary_Data [file jkae114_supplementary_data.zip › Supplemental_Tables_G3-2024-405031.pdf]

Table S1 – Fatty acid composition and content in cultured and *in planta* embryos

		**Fatty acid composition (%)**						
**Sample**	**Fatty acid content (%w/w)**	**C16:0**	**C18:0**	**C18:1**	**C18:2**	**C18:3**	**C20:1-OH**	**C20:2-OH**
Culture-1	23.9513	1.8755	1.6025	13.5180	9.6869	13.5181	57.1292	2.6698
Culture-2	25.2094	1.8542	1.5668	14.8336	10.0005	13.6414	55.6002	2.5033
Culture-3	25.3296	2.1936	1.5644	17.9576	9.2593	14.0703	51.9981	2.9568
in planta-1	23.1815	3.8568	2.9673	15.8807	7.4305	7.9271	60.0077	1.9299
in planta-2	21.3835	4.9979	3.4779	15.5776	7.8856	8.1636	57.8154	2.0820
in planta-3	22.9633	3.7386	3.4961	15.3402	7.8219	8.3867	58.7213	2.4951

Table S2 – Gas chromatography integration of fatty acid methyl esters

Cultured samples

TIC:
Culture-1
20

	Peak #	Ret Time	Area
C16:0	1	3.772	1849700
C17:0 Internal standard	2	4.099	13725940
C18:0	3	4.484	1580504
C18:1	4	4.601	13332278
C18:2	5	4.835	9553832
C18:3	6	5.19	13332399
C20:1-OH	7	13.764	56344401
C20:2-OH	8	15.257	2633095

TIC:
Culture-2
21

	Peak #	Ret Time	Area
C16:0	1	3.758	1990638
C17:0 Internal standard	2	4.084	13308200
C18:0	3	4.472	1682043
C18:1	4	4.588	15924949
C18:2	5	4.82	10736235
C18:3	6	5.177	14645109

In planta samples

TIC:
in_planta-1
Sample-14

	Peak #	Ret Time	Area
C16:0	1	3.993	2822037
C17:0 Internal standard	2	4.354	11273065
C18:0	3	4.786	2171187
C18:1	4	4.92	11620157
C18:2	5	5.184	5437012
C18:3	6	5.589	5800375
C20:1-OH	7	15.417	43908463
C20:2-OH	8	17.123	1412141

TIC:
in_planta-2
Sample-15

	Peak #	Ret Time	Area
C16:0	1	3.999	2625235
C17:0 Internal standard	2	4.362	11165420
C18:0	3	4.789	1826787
C18:1	4	4.928	8182317
C18:2	5	5.192	4142011
C18:3	6	5.597	4288016

C20:1-OH	7	13.747	59690969
C20:2-OH	8	15.229	2687479

C20:1-OH	7	15.42	30368274
C20:2-OH	8	17.17	1093601

TIC:
Culture-3
22

	Peak #	Ret Time	Area
C16:0	1	3.758	2585278
C17:0 Internal standard	2	4.084	13684737
C18:0	3	4.472	1843650
C18:1	4	4.588	21163695
C18:2	5	4.82	10912383
C18:3	6	5.176	16582420
C20:1-OH	7	13.747	61281700
C20:2-OH	8	15.242	3484683

TIC:
in_planta-3
Sample-16

	Peak #	Ret Time	Area
C16:0	1	4.006	2183105
C17:0 Internal standard	2	4.372	11558578
C18:0	3	4.801	2041506
C18:1	4	4.935	8957584
C18:2	5	5.203	4567462
C18:3	6	5.609	4897274
C20:1-OH	7	15.434	34289115
C20:2-OH	8	17.144	1456967

Table S3 – Intermediate mitochondrial assembly results

SPADES k-mer size test

k	Longest contig	sum
33	19531	210387
39	19556	207349
45	27151	204817
55	27183	204793
61	22540	204955
65	22540	205802
67	22510	203958
73	22510	204856
75	22510	206117
79	22510	206316
81	22510	205557
87	27125	203911
95	18998	200207

Processing steps – SPADES k=55 base assembly

Step	Tool	total	longest
**1**	SPADES k=55	204793	27183
**2**	LRSCAF - using super-reads	207185	27183
**3**	L_RNA_scaffolder	208085	34475
**4**	P_RNA_scaffolder	219906	51048
**5**	PEP_scaffolder	219906	85261
**6**	TGS-GC - using super-reads	221320	85774
**7**	MAC2.0, merged with GetOrganelle assembly at k=85	254792	71907
**8**	LRSCAF - using super-reads	254707	71907
**9**	L_RNA_scaffolder	255107	195978
**Final**	P_RNA_scaffolder	255307	255307

Table S4 – Intermediate nuclear genome results

Step	Tool/Assembly	n	N50	sum	gaps	N_count	%N	% top 6	top 6 longest contigs					
**De novo assemblies, initial**	NCBI baseline	9.99E+04	5.91E+03	3.31E+08	1.10E+05	1.62E+06	0.5	0.2	1.26E+05	1.16E+05	1.14E+05	1.06E+05	1.05E+05	9.99E+04
	ABySS k66	1.41E+05	2.78E+03	2.33E+08	2.23E+04	6.03E+05	0.3	0.2	8.79E+04	7.53E+04	7.41E+04	7.41E+04	7.38E+04	7.04E+04
	ABySS k72	1.60E+05	2.53E+03	2.46E+08	3.27E+04	8.25E+05	0.3	0.2	9.72E+04	9.58E+04	7.56E+04	7.42E+04	7.10E+04	6.18E+04
	ABySS MAC	1.16E+05	3.70E+03	2.37E+08	2.23E+04	6.04E+05	0.3	0.2	1.11E+05	1.01E+05	9.79E+04	8.79E+04	8.78E+04	7.54E+04
	SOAP k63	2.37E+05	1.14E+03	2.54E+08	1.11E+05	1.69E+06	0.7	0.1	4.39E+04	4.38E+04	3.86E+04	3.52E+04	3.47E+04	3.47E+04
	SOAP k71	2.38E+05	9.58E+02	2.29E+08	1.02E+05	1.50E+06	0.7	0.1	4.22E+04	3.39E+04	2.91E+04	2.69E+04	2.63E+04	2.61E+04
	SOAP MAC	2.37E+05	1.14E+03	2.54E+08	1.11E+05	1.69E+06	0.7	0.1	4.39E+04	4.39E+04	4.38E+04	3.86E+04	3.52E+04	3.47E+04
	MaSuRCA k67 (auto)	2.83E+05	1.30E+03	3.31E+08	1.77E+05	3.50E+06	1.1	0.1	6.66E+04	6.08E+04	6.01E+04	5.50E+04	5.39E+04	5.34E+04
	MaSuRCA k49	2.26E+05	1.55E+03	2.95E+08	2.27E+05	5.04E+06	1.7	0.1	4.06E+04	3.87E+04	3.80E+04	3.49E+04	3.26E+04	3.23E+04
	MaSuRCA k75	2.77E+05	1.06E+03	2.84E+08	1.81E+05	4.27E+06	1.5	0.1	5.22E+04	4.39E+04	4.10E+04	3.53E+04	3.53E+04	3.41E+04
	MaSurCA MAC	1.40E+05	3.98E+03	3.43E+08	1.76E+05	3.46E+06	1.0	0.2	1.19E+05	1.18E+05	1.00E+05	9.90E+04	7.98E+04	7.63E+04
	MAC all	1.03E+05	6.98E+03	3.51E+08	1.62E+05	3.18E+06	0.9	0.2	1.50E+05	1.48E+05	1.28E+05	1.27E+05	1.19E+05	1.19E+05
**a**	PfGen	9.99E+04	5.91E+03	3.31E+08	1.10E+05	1.62E+06	0.5	0.2	1.26E+05	1.16E+05	1.14E+05	1.06E+05	1.05E+05	9.99E+04
**b**	MAC all	1.03E+05	6.98E+03	3.51E+08	1.62E+05	3.18E+06	0.9	0.2	1.50E+05	1.48E+05	1.28E+05	1.27E+05	1.19E+05	1.19E+05
**c**	LRSCAF	9.94E+04	7.77E+03	3.62E+08	1.71E+05	3.71E+06	1.0	0.2	1.50E+05	1.48E+05	1.42E+05	1.29E+05	1.28E+05	1.27E+05
**d**	SCUBAT	9.80E+04	8.02E+03	3.62E+08	1.72E+05	3.73E+06	1.0	0.3	1.62E+05	1.59E+05	1.50E+05	1.48E+05	1.45E+05	1.45E+05
**e**	PEP_scaffolder	9.70E+04	8.22E+03	3.62E+08	1.73E+05	3.83E+06	1.1	0.3	1.98E+05	1.91E+05	1.90E+05	1.63E+05	1.54E+05	1.50E+05
**f**	L_RNA_scaffolder	9.53E+04	8.61E+03	3.62E+08	1.75E+05	4.00E+06	1.1	0.3	2.31E+05	2.18E+05	1.97E+05	1.90E+05	1.82E+05	1.70E+05
**g**	P_RNA_scaffolder	9.10E+04	9.82E+03	3.63E+08	1.79E+05	4.45E+06	1.2	0.4	3.10E+05	2.47E+05	2.43E+05	2.37E+05	2.26E+05	2.18E+05
**h**	TGS-GC	9.10E+04	9.79E+03	3.62E+08	1.60E+05	3.98E+06	1.1	0.4	3.08E+05	2.46E+05	2.42E+05	2.38E+05	2.25E+05	2.18E+05
**i**	GMCloser	8.95E+04	9.87E+03	3.61E+08	1.35E+05	5.57E+06	1.5	0.4	3.08E+05	2.46E+05	2.42E+05	2.38E+05	2.25E+05	2.18E+05
**j**	Purge Haplotigs	3.47E+04	1.44E+04	2.69E+08	9.57E+04	4.24E+06	1.6	0.5	3.08E+05	2.46E+05	2.42E+05	2.38E+05	2.25E+05	2.18E+05
**k**	RagTag scaffold	3.22E+04	1.68E+04	2.69E+08	9.82E+04	4.50E+06	1.7	0.6	3.08E+05	2.64E+05	2.52E+05	2.46E+05	2.42E+05	2.25E+05
**l**	LRSCAF	3.15E+04	1.72E+04	2.70E+08	9.87E+04	4.56E+06	1.7	0.6	3.08E+05	2.64E+05	2.52E+05	2.46E+05	2.42E+05	2.29E+05
**m**	SCUBAT	3.04E+04	1.85E+04	2.70E+08	9.98E+04	4.57E+06	1.7	0.7	5.47E+05	3.08E+05	3.04E+05	3.00E+05	2.58E+05	2.46E+05
**n**	PEP_scaffolder	2.99E+04	1.92E+04	2.70E+08	1.00E+05	4.62E+06	1.7	0.9	5.47E+05	4.46E+05	3.93E+05	3.92E+05	3.26E+05	3.07E+05

**o**	L_RNA_scaffolder	2.92E+04	2.04E+04	2.70E+08	1.01E+05	4.69E+06	1.7	1.1	6.92E+05	5.49E+05	4.46E+05	4.13E+05	4.08E+05	3.80E+05
**p**	P_RNA_scaffolder	2.73E+04	2.47E+04	2.70E+08	1.03E+05	4.90E+06	1.8	1.3	8.41E+05	5.79E+05	5.60E+05	5.32E+05	5.26E+05	5.18E+05
**q**	TGS-GC	2.73E+04	2.47E+04	2.70E+08	1.01E+05	4.82E+06	1.8	1.3	8.39E+05	5.78E+05	5.59E+05	5.32E+05	5.25E+05	5.17E+05
**r**	Purge Haplotigs	2.61E+04	2.52E+04	2.66E+08	9.92E+04	4.73E+06	1.8	1.3	8.39E+05	5.78E+05	5.59E+05	5.32E+05	5.25E+05	5.17E+05
**s**	RagTag scaffold	2.59E+04	2.58E+04	2.66E+08	9.95E+04	4.75E+06	1.8	1.3	8.42E+05	5.78E+05	5.59E+05	5.32E+05	5.25E+05	5.17E+05
**t**	LRSCAF	2.59E+04	2.59E+04	2.67E+08	9.96E+04	4.76E+06	1.8	1.3	8.42E+05	5.78E+05	5.59E+05	5.32E+05	5.25E+05	5.17E+05
**u**	SCUBAT	2.56E+04	2.71E+04	2.67E+08	9.98E+04	4.76E+06	1.8	1.4	8.42E+05	6.05E+05	5.78E+05	5.59E+05	5.32E+05	5.25E+05
**v**	PEP_scaffolder	2.55E+04	2.75E+04	2.67E+08	9.99E+04	4.77E+06	1.8	1.4	8.42E+05	6.05E+05	5.78E+05	5.59E+05	5.47E+05	5.32E+05
**w**	L_RNA_scaffolder	2.51E+04	2.93E+04	2.67E+08	1.00E+05	4.80E+06	1.8	1.4	8.42E+05	6.05E+05	5.78E+05	5.59E+05	5.57E+05	5.54E+05
**x**	P_RNA_scaffolder	2.42E+04	3.35E+04	2.67E+08	1.01E+05	4.89E+06	1.8	1.7	1.02E+06	8.80E+05	6.66E+05	6.52E+05	6.39E+05	6.35E+05
**y**	TGS-GC	2.42E+04	3.36E+04	2.67E+08	8.78E+04	4.44E+06	1.7	1.7	1.03E+06	8.84E+05	6.65E+05	6.53E+05	6.41E+05	6.36E+05
**A**	Chromosemble	1.01E+04	4.20E+07	2.73E+08	1.02E+05	9.99E+06	3.7	90.4	6.21E+07	5.85E+07	4.20E+07	3.34E+07	2.61E+07	2.48E+07
**B**	LRSCAF	1.01E+04	4.20E+07	2.73E+08	1.02E+05	9.99E+06	3.7	90.4	6.21E+07	5.85E+07	4.20E+07	3.34E+07	2.61E+07	2.48E+07
**C**	PEP_scaffolder	1.01E+04	4.20E+07	2.73E+08	1.02E+05	9.99E+06	3.7	90.4	6.21E+07	5.85E+07	4.20E+07	3.34E+07	2.61E+07	2.48E+07
**D**	L_RNA_scaffolder	1.01E+04	4.20E+07	2.73E+08	1.02E+05	9.99E+06	3.7	90.4	6.21E+07	5.85E+07	4.20E+07	3.34E+07	2.61E+07	2.48E+07
**E**	P_RNA_scaffolder	1.01E+04	4.20E+07	2.73E+08	1.02E+05	9.99E+06	3.7	90.4	6.21E+07	5.85E+07	4.20E+07	3.34E+07	2.61E+07	2.48E+07
**F**	TGS-GC	1.01E+04	4.19E+07	2.73E+08	1.01E+05	9.96E+06	3.7	90.4	6.20E+07	5.85E+07	4.19E+07	3.33E+07	2.60E+07	2.47E+07

Table S5 – Configuration files for MAKER

MAKER Round 1

#-----Genome (these are always required)
genome=pseudochromosomes.fasta #genome sequence (fasta file or fasta embeded in GFF3 file)
organism_type=eukaryotic #eukaryotic or prokaryotic. Default is eukaryotic

#-----Re-annotation Using MAKER Derived GFF3
maker_gff= #MAKER derived GFF3 file
est_pass=0 #use ESTs in maker_gff: 1 = yes, 0 = no
altest_pass=0 #use alternate organism ESTs in maker_gff: 1 = yes, 0 = no
protein_pass=0 #use protein alignments in maker_gff: 1 = yes, 0 = no
rm_pass=0 #use repeats in maker_gff: 1 = yes, 0 = no
model_pass=0 #use gene models in maker_gff: 1 = yes, 0 = no
pred_pass=0 #use ab-initio predictions in maker_gff: 1 = yes, 0 = no
other_pass=0 #passthrough anyything else in maker_gff: 1 = yes, 0 = no

#-----EST Evidence (for best results provide a file for at least one)
est=.../de_novo_assembly.mRNA.fasta,/.../cleaned_TAIR_rna.fasta #set of ESTs or assembled mRNA-seq in fasta format
altest= #EST/cDNA sequence file in fasta format from an alternate organism
est_gff= #aligned ESTs or mRNA-seq from an external GFF3 file
altest_gff= #aligned ESTs from a closly relate species in GFF3 format

#-----Protein Homology Evidence (for best results provide a file for at least one)
protein=/.../Brassicaceae5.faa #protein sequence file in fasta format (i.e. from mutiple organisms)
protein_gff= #aligned protein homology evidence from an external GFF3 file

#-----Repeat Masking (leave values blank to skip repeat masking)
model_org= #select a model organism for RepBase masking in RepeatMasker
rmlib=/.../PhyfeV1_allRepeats.lib #provide an organism specific repeat library in fasta format for RepeatMasker

repeat_protein=/.../Tpases020812.faa #provide a fasta file of transposable element proteins for RepeatRunner
rm_gff= #pre-identified repeat elements from an external GFF3 file
prok_rm=0 #forces MAKER to repeatmask prokaryotes (no reason to change this), 1 = yes, 0 = no
softmask=1 #use soft-masking rather than hard-masking in BLAST (i.e. seg and dust filtering)

#-----Gene Prediction
snaphmm= #SNAP HMM file
gmhmm= #GeneMark HMM file
augustus_species= #Augustus gene prediction species model
fgenesh_par_file= #FGENESH parameter file
pred_gff= #ab-initio predictions from an external GFF3 file
model_gff= #annotated gene models from an external GFF3 file (annotation pass-through)
run_evm=0 #run EvidenceModeler, 1 = yes, 0 = no
est2genome=1 #infer gene predictions directly from ESTs, 1 = yes, 0 = no
protein2genome=1 #infer predictions from protein homology, 1 = yes, 0 = no
trna=0 #find tRNAs with tRNAscan, 1 = yes, 0 = no
snoscan_rrna= #rRNA file to have Snoscan find snoRNAs
snoscan_meth= #-O-methylation site fileto have Snoscan find snoRNAs
unmask=0 #also run ab-initio prediction programs on unmasked sequence, 1 = yes, 0 = no
allow_overlap= #allowed gene overlap fraction (value from 0 to 1, blank for default)

#-----Other Annotation Feature Types (features MAKER doesn't recognize)
other_gff= #extra features to pass-through to final MAKER generated GFF3 file

#-----External Application Behavior Options
alt_peptide=C #amino acid used to replace non-standard amino acids in BLAST databases
cpus=64 #max number of cpus to use in BLAST and RepeatMasker (not for MPI, leave 1 when using MPI)

#-----MAKER Behavior Options
max_dna_len=100000 #length for dividing up contigs into chunks (increases/decreases memory usage)

min_contig=1 #skip genome contigs below this length (under 10kb are often useless)

pred_flank=200 #flank for extending evidence clusters sent to gene predictors
pred_stats=0 #report AED and QI statistics for all predictions as well as models
AED_threshold=1 #Maximum Annotation Edit Distance allowed (bound by 0 and 1)
min_protein=0 #require at least this many amino acids in predicted proteins
alt_splice=0 #Take extra steps to try and find alternative splicing, 1 = yes, 0 = no
always_complete=0 #extra steps to force start and stop codons, 1 = yes, 0 = no
map_forward=0 #map names and attributes forward from old GFF3 genes, 1 = yes, 0 = no
keep_preds=0 #Concordance threshold to add unsupported gene prediction (bound by 0 and 1)

split_hit=10000 #length for the splitting of hits (expected max intron size for evidence alignments)
min_intron=20 #minimum intron length (used for alignment polishing)
single_exon=0 #consider single exon EST evidence when generating annotations, 1 = yes, 0 = no
single_length=250 #min length required for single exon ESTs if 'single_exon is enabled'
correct_est_fusion=0 #limits use of ESTs in annotation to avoid fusion genes

tries=2 #number of times to try a contig if there is a failure for some reason
clean_try=0 #remove all data from previous run before retrying, 1 = yes, 0 = no
clean_up=1 #removes theVoid directory with individual analysis files, 1 = yes, 0 = no
TMP= #specify a directory other than the system default temporary directory for temporary files

MAKER Round 2

#-----Genome (these are always required)
genome=pseudochromosomes.fasta #genome sequence (fasta file or fasta embeded in GFF3 file)
organism_type=eukaryotic #eukaryotic or prokaryotic. Default is eukaryotic

```
#-----Re-annotation Using MAKER Derived GFF3
maker_gff= #MAKER derived GFF3 file
est_pass=0 #use ESTs in maker_gff: 1 = yes, 0 = no
altest_pass=0 #use alternate organism ESTs in maker_gff: 1 = yes, 0 = no
protein_pass=0 #use protein alignments in maker_gff: 1 = yes, 0 = no
rm_pass=0 #use repeats in maker_gff: 1 = yes, 0 = no
model_pass=0 #use gene models in maker_gff: 1 = yes, 0 = no
pred_pass=0 #use ab-initio predictions in maker_gff: 1 = yes, 0 = no
other_pass=0 #passthrough anyything else in maker_gff: 1 = yes, 0 = no

#-----EST Evidence (for best results provide a file for at least one)
est= #set of ESTs or assembled mRNA-seq in fasta format
altest= #EST/cDNA sequence file in fasta format from an alternate organism
est_gff=/.../Phyfe_rnd1.all.maker.est2genome.gff #aligned ESTs or mRNA-seq from an external GFF3 file
altest_gff= #aligned ESTs from a closly relate species in GFF3 format

#-----Protein Homology Evidence (for best results provide a file for at least one)
protein=  #protein sequence file in fasta format (i.e. from mutiple organisms)
protein_gff=/.../Phyfe_rnd1.all.maker.protein2genome.gff  #aligned protein homology evidence from an external GFF3 file

#-----Repeat Masking (leave values blank to skip repeat masking)
model_org= #select a model organism for RepBase masking in RepeatMasker
rmlib= #provide an organism specific repeat library in fasta format for RepeatMasker
repeat_protein=#provide a fasta file of transposable element proteins for RepeatRunner
rm_gff=/.../Phyfe_rnd1.all.maker.repeats.gff #pre-identified repeat elements from an external GFF3 file
prok_rm=0 #forces MAKER to repeatmask prokaryotes (no reason to change this), 1 = yes, 0 = no
softmask=1 #use soft-masking rather than hard-masking in BLAST (i.e. seg and dust filtering)

#-----Gene Prediction
snaphmm=/.../Phyfe_rnd1.zff.hmm #SNAP HMM file
```

```
gmhmm= #GeneMark HMM file
augustus_species=P_fendleri #Augustus gene prediction species model
fgenesh_par_file= #FGENESH parameter file
pred_gff= #ab-initio predictions from an external GFF3 file
model_gff= #annotated gene models from an external GFF3 file (annotation pass-through)
run_evm=0 #run EvidenceModeler, 1 = yes, 0 = no
est2genome=0 #infer gene predictions directly from ESTs, 1 = yes, 0 = no
protein2genome=0 #infer predictions from protein homology, 1 = yes, 0 = no
trna=0 #find tRNAs with tRNAscan, 1 = yes, 0 = no
snoscan_rrna= #rRNA file to have Snoscan find snoRNAs
snoscan_meth= #-O-methylation site fileto have Snoscan find snoRNAs
unmask=0 #also run ab-initio prediction programs on unmasked sequence, 1 = yes, 0 = no
allow_overlap= #allowed gene overlap fraction (value from 0 to 1, blank for default)

#-----Other Annotation Feature Types (features MAKER doesn't recognize)
other_gff= #extra features to pass-through to final MAKER generated GFF3 file

#-----External Application Behavior Options
alt_peptide=C #amino acid used to replace non-standard amino acids in BLAST databases
cpus=1 #max number of cpus to use in BLAST and RepeatMasker (not for MPI, leave 1 when using MPI)

#-----MAKER Behavior Options
max_dna_len=100000 #length for dividing up contigs into chunks (increases/decreases memory usage)
min_contig=1 #skip genome contigs below this length (under 10kb are often useless)

pred_flank=200 #flank for extending evidence clusters sent to gene predictors
pred_stats=0 #report AED and QI statistics for all predictions as well as models
AED_threshold=1 #Maximum Annotation Edit Distance allowed (bound by 0 and 1)
min_protein=0 #require at least this many amino acids in predicted proteins
alt_splice=0 #Take extra steps to try and find alternative splicing, 1 = yes, 0 = no
```

always_complete=0 #extra steps to force start and stop codons, 1 = yes, 0 = no
map_forward=0 #map names and attributes forward from old GFF3 genes, 1 = yes, 0 = no
keep_preds=0 #Concordance threshold to add unsupported gene prediction (bound by 0 and 1)

split_hit=10000 #length for the splitting of hits (expected max intron size for evidence alignments)
min_intron=20 #minimum intron length (used for alignment polishing)
single_exon=0 #consider single exon EST evidence when generating annotations, 1 = yes, 0 = no
single_length=250 #min length required for single exon ESTs if 'single_exon is enabled'
correct_est_fusion=0 #limits use of ESTs in annotation to avoid fusion genes

tries=2 #number of times to try a contig if there is a failure for some reason
clean_try=0 #remove all data from previous run before retrying, 1 = yes, 0 = no
clean_up=0 #removes theVoid directory with individual analysis files, 1 = yes, 0 = no
TMP= #specify a directory other than the system default temporary directory for temporary files

MAKER Round 3
#-----Genome (these are always required)
genome=pseudochromosomes.fasta #genome sequence (fasta file or fasta embeded in GFF3 file)
organism_type=eukaryotic #eukaryotic or prokaryotic. Default is eukaryotic

#-----Re-annotation Using MAKER Derived GFF3
maker_gff= #MAKER derived GFF3 file
est_pass=0 #use ESTs in maker_gff: 1 = yes, 0 = no
altest_pass=0 #use alternate organism ESTs in maker_gff: 1 = yes, 0 = no
protein_pass=0 #use protein alignments in maker_gff: 1 = yes, 0 = no
rm_pass=0 #use repeats in maker_gff: 1 = yes, 0 = no
model_pass=0 #use gene models in maker_gff: 1 = yes, 0 = no

```
pred_pass=0 #use ab-initio predictions in maker_gff: 1 = yes, 0 = no
other_pass=0 #passthrough anyything else in maker_gff: 1 = yes, 0 = no

#-----EST Evidence (for best results provide a file for at least one)
est= #set of ESTs or assembled mRNA-seq in fasta format
altest= #EST/cDNA sequence file in fasta format from an alternate organism
est_gff=/.../Phyfe_rnd1.all.maker.est2genome.gff #aligned ESTs or mRNA-seq from an external GFF3 file
altest_gff= #aligned ESTs from a closly relate species in GFF3 format

#-----Protein Homology Evidence (for best results provide a file for at least one)
protein=  #protein sequence file in fasta format (i.e. from mutiple organisms)
protein_gff=/.../Phyfe_rnd1.all.maker.protein2genome.gff  #aligned protein homology evidence from an external GFF3 file

#-----Repeat Masking (leave values blank to skip repeat masking)
model_org= #select a model organism for RepBase masking in RepeatMasker
rmlib= #provide an organism specific repeat library in fasta format for RepeatMasker
repeat_protein=#provide a fasta file of transposable element proteins for RepeatRunner
rm_gff=/.../Phyfe_rnd1.all.maker.repeats.gff #pre-identified repeat elements from an external GFF3 file
prok_rm=0 #forces MAKER to repeatmask prokaryotes (no reason to change this), 1 = yes, 0 = no
softmask=1 #use soft-masking rather than hard-masking in BLAST (i.e. seg and dust filtering)

#-----Gene Prediction
snaphmm=/.../Phyfe_rnd2.zff.hmm #SNAP HMM file
gmhmm= #GeneMark HMM file
augustus_species=P_fendleri #Augustus gene prediction species model
fgenesh_par_file= #FGENESH parameter file
pred_gff= #ab-initio predictions from an external GFF3 file
model_gff= #annotated gene models from an external GFF3 file (annotation pass-through)
run_evm=0 #run EvidenceModeler, 1 = yes, 0 = no
est2genome=0 #infer gene predictions directly from ESTs, 1 = yes, 0 = no
```

protein2genome=0 #infer predictions from protein homology, 1 = yes, 0 = no
trna=0 #find tRNAs with tRNAscan, 1 = yes, 0 = no
snoscan_rrna= #rRNA file to have Snoscan find snoRNAs
snoscan_meth= #-O-methylation site fileto have Snoscan find snoRNAs
unmask=0 #also run ab-initio prediction programs on unmasked sequence, 1 = yes, 0 = no
allow_overlap= #allowed gene overlap fraction (value from 0 to 1, blank for default)

#-----Other Annotation Feature Types (features MAKER doesn't recognize)
other_gff= #extra features to pass-through to final MAKER generated GFF3 file

#-----External Application Behavior Options
alt_peptide=C #amino acid used to replace non-standard amino acids in BLAST databases
cpus=1 #max number of cpus to use in BLAST and RepeatMasker (not for MPI, leave 1 when using MPI)

#-----MAKER Behavior Options
max_dna_len=100000 #length for dividing up contigs into chunks (increases/decreases memory usage)
min_contig=1 #skip genome contigs below this length (under 10kb are often useless)

pred_flank=200 #flank for extending evidence clusters sent to gene predictors
pred_stats=0 #report AED and QI statistics for all predictions as well as models
AED_threshold=1 #Maximum Annotation Edit Distance allowed (bound by 0 and 1)
min_protein=0 #require at least this many amino acids in predicted proteins
alt_splice=0 #Take extra steps to try and find alternative splicing, 1 = yes, 0 = no
always_complete=0 #extra steps to force start and stop codons, 1 = yes, 0 = no
map_forward=0 #map names and attributes forward from old GFF3 genes, 1 = yes, 0 = no
keep_preds=0 #Concordance threshold to add unsupported gene prediction (bound by 0 and 1)

split_hit=10000 #length for the splitting of hits (expected max intron size for evidence alignments)
min_intron=20 #minimum intron length (used for alignment polishing)
single_exon=0 #consider single exon EST evidence when generating annotations, 1 = yes, 0 = no

single_length=250 #min length required for single exon ESTs if 'single_exon is enabled'
correct_est_fusion=0 #limits use of ESTs in annotation to avoid fusion genes

tries=2 #number of times to try a contig if there is a failure for some reason
clean_try=0 #remove all data from previous run before retrying, 1 = yes, 0 = no
clean_up=0 #removes theVoid directory with individual analysis files, 1 = yes, 0 = no
TMP= #specify a directory other than the system default temporary directory for temporary files

Table S6 – Intermediate transcriptome assembly results

Prefilter Test

**Filter**	**Total sequences**	**% BLASTx Hits >80% Alignment**	**Mean sequence length**	**Predicted genes**	**Single Copy**	**Duplicate**	**Fragmented**	**Missing**	**Read Representation (%)**
**None**	199563	66.6085	1124	113133	10.7	75.9	2	11.4	93.55
**CD-HIT-EST**	135323	67.6105	1091	68423	25.5	61.1	2.1	11.3	91.25
**CD-HIT-EST->tr2aacds**	71370	73.8089	1464	59584	27.7	58.5	2	11.8	88.5
**CD-HIT-EST->tr2aacds->LONG**	33347	74.6760	1292	29335	72.4	12.6	2.3	12.7	75.16
**tr2aacds**	106359	72.7879	1412	91014	12.8	73.6	2	11.6	91.75
**tr2aacds→CD-HIT-EST***	70840	73.9294	1450	57783	28.2	58.1	2	11.7	88.53
**tr2aacds->CD-HIT-EST->LONG**	27133	68.4516	1195	22775	55.9	7	2.6	34.5	65.57
**tr2aacds->LONG**	37100	74.5590	1203	32071	69.5	15.3	2.5	12.7	79.41

Notes:
tr2aacds: default pipeline from EvidentialGene
CD-HIT-EST: run using 0.98 identity cutoff
LONG: only longest variant of each transcript kept

***Chosen pre-filter**

Post-filter test

**Filter**	**Total sequences**	**% BLASTx Hits >80% Alignment**	**Mean sequence length**	**Predicted genes**	**Single Copy**	**Duplicate**	**Fragmented**	**Missing**	**Read Representation (%)**
**T**	95316	75.9094	1539	75636	21	67.5	0.9	10.6	92.31
**T->C**	82834	75.8307	1565	64887	25.7	62.8	0.9	10.6	91.48

Notes:
T: transcripts

										under 1 TPM (cumulative) removed
**T→C→c***	74416	77.9028	1661	61766	25.7	62.1	0.9	11.3	91.39	*C: CD-HIT-EST run using 0.95 identity cutoff*
**Final following Trinotate**	75096									*c: Yang & Smith 2013 chimera removal method*

***Chosen filtered assembly**

Table S7– BUSCO results using embryophyta_odb10 database in genome mode, including organellular genomes and chromosomes

**Complete**	**Single**	**Duplicate**	**Fragment**	**Missing**
77.1	71.7	5.4	12.3	10.6

MAKER Annotation Results

**gene models**	**Avg. gene length**	**Gene models with AED < 0.5**
39859	2009.39	0.873

Table S8 – Coordinates for Circos links presented in synteny analysis

	Physaria fendleri intraspecies links (Figure 5)						***Physaria fendleri interspecies links (Figure 6)***				
**chr2**	35769716	53841626	**chr3**	18095450	40190748	**chr14**	9474076	9475246	**chr1**	4635507	4636672
**chr1**	17657999	35325158	**chr1**	41249390	35520673	**chr16**	206991	209582	**chr2**	13429350	13426688
**chr2**	31414404	48696030	**chr6**	12955107	22148268	**chr13**	27948979	27951270	**chr1**	56991336	56988994
**chr3**	20995839	35264513	**chr3**	32194071	41110333	**chr11**	21258278	21259399	**chr5**	15544321	15545558
**chr2**	41473827	54046048	**chr4**	21289698	11502565	**chr7**	9021652	9023034	**chr1**	8545814	8547196
**chr2**	23663877	35108114	**chr2**	48134884	36251432	**chr17**	16838769	16840612	**chr2**	12602233	12600390
**chr1**	10846876	19718281	**chr5**	6707726	1657107	**chr17**	19053838	19055441	**chr2**	29899197	29900800
**chr1**	2468831	10003829	**chr1**	48440045	61367097	**chr11**	25212655	25214078	**chr5**	7990098	7988611
**chr2**	6283912	12603442	**chr6**	13975558	1608622	**chr12**	393919	396155	**chr6**	2234051	2236332
**chr1**	20298415	26220353	**chr2**	33210451	30831490	**chr13**	26389979	26392317	**chr6**	13015168	13017570
**chr1**	1038681	6887499	**chr2**	50175754	45667126	**chr17**	2673471	2674625	**chr2**	34171774	34172928
**chr1**	30160860	35680013	**chr5**	21470681	16160174	**chr8**	33868973	33870550	**chr2**	27456970	27458550
**chr1**	7407987	11762814	**chr2**	12103030	8733904	**chr7**	2476171	2477981	**chr1**	23224058	23222180
**chr2**	2333758	6544827	**chr5**	17127360	15340059	**chr12**	20637720	20639792	**chr5**	5521264	5518684
**chr3**	4097509	8026123	**chr6**	11562418	16040636	**chr16**	777134	779270	**chr2**	41484952	41487092
**chr2**	27981746	30865334	**chr3**	36913768	28815886	**chr15**	2182104	2183911	**chr1**	23224055	23222075
**chr1**	26262570	28924350	**chr2**	1793652	923996	**chr12**	1059413	1060586	**chr6**	3193707	3194880
**chr4**	10238823	12693894	**chr5**	19163064	18146981	**chr8**	5801692	5803219	**chr2**	46540586	46542465
**chr2**	12026597	14410231	**chr2**	17165672	14569852	**chr8**	33516863	33517986	**chr2**	48733085	48734208
**chr2**	10545182	12603442	**chr4**	9666517	12118769	**chr17**	2745161	2747397	**chr2**	34305470	34308096
**chr4**	11489717	13546730	**chr4**	13990276	13134704	**chr14**	1870588	1872825	**chr3**	13117714	13115454
**chr1**	44732514	46476750	**chr4**	28131248	26925728	**chr11**	30074400	30076779	**chr2**	39882467	39884854
**chr5**	23121780	24619756	**chr6**	24553488	21070054	**chr13**	10513266	10515364	**chr5**	5334707	5336894
**chr2**	3966568	5270690	**chr2**	34482835	36001774	**chr10**	5018699	5021277	**chr4**	14446593	14449177
**chr1**	49727130	50930711	**chr2**	56349951	57006193	**chr12**	22420722	22421910	**chr6**	20003679	20004864
**chr1**	45360517	46432181	**chr2**	1737259	2555682	**chr13**	27217680	27219376	**chr4**	1067361	1069056
**chr1**	52020869	53063283	**chr6**	14296647	14725015	**chr7**	463570	465927	**chr1**	38289308	38286884
**chr2**	24160631	25202191	**chr6**	11644485	12437332	**chr8**	19161354	19163176	**chr2**	14472491	14474313
**chr1**	60122927	61139599	**chr2**	740166	1216049	**chr10**	4351396	4352669	**chr4**	13155678	13154405
**chr3**	38792298	39796808	**chr4**	1946620	1222505	**chr17**	5825083	5826764	**chr2**	48228637	48230400
**chr1**	6961067	7465511	**chr6**	18670628	19678432	**chr10**	30033608	30035732	**chr4**	25892786	25894912

**chr15**	6207662	6208857	**chr6**	3608577	3609839
**chr8**	7939150	7940427	**chr2**	35194523	35193171
**chr7**	6536187	6538108	**chr5**	13808389	13806045
**chr15**	3484356	3485532	**chr1**	28673098	28674382
**chr16**	15404219	15406021	**chr1**	22472883	22474752
**chr13**	719460	721854	**chr2**	39884853	39882458
**chr17**	7763002	7764069	**chr2**	58249217	58250396
**chr17**	22497401	22498531	**chr6**	20004865	20005996
**chr7**	7878804	7880562	**chr4**	11278571	11280384
**chr11**	17162299	17164510	**chr5**	5336889	5334589
**chr13**	8064550	8066175	**chr4**	31811727	31810102
**chr17**	18087372	18090108	**chr1**	52862673	52859922
**chr8**	5596181	5597992	**chr2**	43249663	43247783
**chr8**	4768709	4771189	**chr2**	36694940	36697419
**chr13**	29104533	29106725	**chr3**	10999952	10997760
**chr7**	10346585	10348481	**chr1**	5279612	5277654
**chr7**	1025683	1026881	**chr1**	36977396	36978688
**chr15**	5952434	5953705	**chr1**	54837773	54839044
**chr14**	3448253	3449886	**chr2**	52414960	52413266
**chr16**	1038158	1039753	**chr2**	1382853	1384448
**chr16**	16962497	16964232	**chr1**	28556912	28553907
**chr7**	1362753	1364295	**chr5**	16125951	16124409
**chr13**	10760493	10762262	**chr6**	5693439	5691496
**chr7**	7198827	7200095	**chr1**	54837782	54839050
**chr8**	5240907	5242270	**chr2**	39014399	39012976
**chr15**	570440	571500	**chr1**	38644700	38645760
**chr8**	6235342	6237006	**chr2**	55015202	55013406
**chr14**	7797160	7798694	**chr2**	8169523	8171057
**chr15**	20190338	20192146	**chr3**	17483816	17485824
**chr13**	20968206	20970025	**chr1**	864976	866925
**chr8**	36047685	36049286	**chr2**	29900799	29899198
**chr11**	18745451	18746717	**chr5**	13021919	13023185
**chr13**	5120366	5122110	**chr5**	7988644	7990519
**chr8**	7339236	7340452	**chr2**	48806714	48807936

**chr7**	2120923	2123123	**chr2**	538851	536576
**chr17**	16345157	16347686	**chr4**	14449125	14446592
**chr7**	3998341	3999484	**chr1**	28673102	28674353
**chr16**	17907211	17909246	**chr6**	879385	877227
**chr8**	2492017	2493229	**chr2**	32620545	32621828
**chr8**	20319029	20320972	**chr2**	41487092	41485149
**chr14**	7795799	7796910	**chr2**	8168149	8169262
**chr15**	380688	383666	**chr1**	38906798	38903808
**chr14**	7786959	7789231	**chr2**	8159233	8161526
**chr16**	14461245	14463781	**chr1**	8548351	8545815
**chr15**	908653	909849	**chr1**	36977398	36978688
**chr11**	30078543	30080412	**chr2**	39885913	39887858
**chr10**	11824987	11826932	**chr1**	56991349	56989356
**chr13**	4396509	4397905	**chr1**	32466971	32468484
**chr11**	27595713	27596801	**chr5**	23716214	23717507
**chr15**	432437	433808	**chr1**	38301695	38300323
**chr17**	25642836	25644012	**chr6**	23765248	23766424
**chr12**	9640202	9641259	**chr6**	15552513	15553567
**chr13**	22177693	22179637	**chr4**	7002080	7000142
**chr16**	15285109	15286896	**chr4**	11280383	11278532
**chr14**	19653211	19655231	**chr3**	168695	166614
**chr9**	5781156	5782497	**chr3**	32041313	32039972
**chr13**	29901659	29903288	**chr1**	35921921	35923568
**chr9**	41729	43259	**chr3**	166614	168211
**chr8**	26266218	26267648	**chr2**	8160108	8161538
**chr14**	9410650	9413294	**chr1**	4669219	4666575
**chr7**	9023056	9024193	**chr1**	8547215	8548352
**chr11**	4354318	4355378	**chr1**	12120065	12121128
**chr13**	1955827	1956913	**chr5**	24814788	24813568
**chr11**	26072630	26074030	**chr1**	32468487	32466970
**chr13**	715809	717677	**chr2**	39887857	39885913
**chr8**	26275485	26277111	**chr2**	8169431	8171057
**chr13**	4790736	4792385	**chr5**	21430229	21428080
**chr16**	23884	25274	**chr4**	12875118	12873728

**chr10**	4297729	4299020	**chr4**	13620926	13622217
**chr10**	5499384	5502556	**chr1**	4672514	4669281
**chr12**	2688855	2690072	**chr1**	401409	402708
**chr8**	2419942	2421219	**chr4**	15640497	15641841
**chr17**	4411877	4414696	**chr2**	39165951	39163138
**chr10**	30126322	30128807	**chr6**	17212568	17215050
**chr10**	4528129	4529948	**chr3**	10999579	10997634
**chr13**	9673219	9674545	**chr6**	24430313	24431777
**chr8**	3163819	3166038	**chr2**	34305486	34308096
**chr13**	332230	333631	**chr2**	3046045	3047447
**chr11**	4438300	4439346	**chr2**	9413654	9412608
**chr16**	6602199	6604849	**chr2**	11879840	11882496
**chr9**	6179269	6180755	**chr2**	15630335	15628849
**chr8**	27006972	27008283	**chr2**	15290369	15291748
**chr14**	9407105	9410559	**chr1**	4672838	4669309
**chr10**	14285164	14287694	**chr4**	15671295	15668765
**chr8**	5097927	5100747	**chr2**	39165951	39163140
**chr11**	18779442	18780722	**chr6**	24431775	24430425
**chr7**	15129835	15131954	**chr1**	55380051	55377776
**chr15**	434724	440023	**chr1**	38299500	38294204
**chr16**	9877022	9878173	**chr2**	23575383	23576576
**chr16**	15269403	15271551	**chr4**	11295441	11293263
**chr7**	459815	463551	**chr1**	38293122	38289389
**chr15**	441064	444778	**chr1**	38293119	38289408
**chr7**	4993515	4994829	**chr1**	19412255	19410944
**chr10**	4296784	4298751	**chr4**	13619981	13622272
**chr13**	25089517	25091204	**chr3**	11407794	11405967
**chr7**	453541	458497	**chr1**	38299450	38294494
**chr14**	3384572	3385771	**chr2**	52502526	52503701
**chr16**	16415112	16416328	**chr1**	402706	401408
**chr14**	3484940	3485993	**chr2**	52471622	52470580
**chr14**	3271699	3272820	**chr3**	29827049	29828148
**chr11**	16711681	16712842	**chr6**	5692151	5693440
**chr14**	17780128	17781394	**chr3**	23280307	23281664

**chr14**	3295718	3297198	**chr2**	41921923	41920381
**chr17**	5101455	5104331	**chr2**	43675778	43678714
**chr8**	5872893	5875762	**chr2**	43675780	43678709
**chr10**	5017104	5018698	**chr4**	14444998	14446592
**chr11**	30794776	30796086	**chr2**	3046136	3047447
**chr10**	1284136	1286074	**chr3**	10727425	10729876
**chr11**	25628899	25630552	**chr5**	21428077	21430230
**chr10**	2037812	2039434	**chr4**	7000065	7001738
**chr14**	3405372	3406480	**chr3**	29856712	29857816
**chr12**	2024829	2026540	**chr1**	28553217	28555319
**chr8**	18535215	18538042	**chr5**	12341249	12344076
**chr17**	16347687	16349316	**chr4**	14446591	14444962
**chr14**	3472051	3473978	**chr3**	29759555	29761517
**chr14**	3328774	3330153	**chr2**	52493929	52492570
**chr14**	3460722	3461781	**chr2**	52486992	52485941
**chr9**	8430145	8431193	**chr2**	38879233	38880309
**chr16**	2539659	2542479	**chr5**	12341249	12344066
**chr14**	3303518	3304722	**chr2**	52125697	52126888
**chr14**	3269898	3271299	**chr3**	29825158	29826640
**chr15**	20542409	20543458	**chr2**	38880309	38879232
**chr14**	3399546	3400757	**chr2**	52531700	52532900
**chr7**	7896748	7898903	**chr4**	11293252	11295437
**chr8**	17382453	17383818	**chr2**	52383386	52381963
**chr14**	3279951	3281956	**chr3**	29835826	29837806
**chr14**	3330093	3331127	**chr2**	41926720	41927744
**chr14**	3412132	3413165	**chr3**	29758454	29757435
**chr14**	3415564	3417546	**chr3**	29754812	29752681
**chr14**	3389370	3390405	**chr2**	52520300	52521393
**chr14**	3297892	3299082	**chr2**	41917231	41916041
**chr14**	3347604	3350528	**chr2**	52493854	52496775
**chr14**	3339202	3341138	**chr2**	41935919	41938119
**chr14**	3393261	3395240	**chr2**	52525254	52527231
**chr14**	3391183	3393311	**chr2**	52523127	52525254
**chr14**	3310871	3312640	**chr2**	52390894	52389129

**chr14**	3299518	3300945	**chr2**	41915399	41913982
**chr14**	3343521	3345118	**chr2**	52438668	52437071
**chr8**	17392334	17393424	**chr2**	52395848	52394756
**chr14**	3421162	3422401	**chr3**	29748867	29747640
**chr14**	3408590	3409874	**chr3**	29847702	29849024
**chr14**	3423499	3424851	**chr3**	29746501	29745148
**chr14**	3362413	3364232	**chr2**	41948144	41950101
**chr14**	3442787	3444309	**chr2**	52385031	52386608
**chr14**	3351422	3353727	**chr2**	52490277	52487920
**chr14**	3335035	3337979	**chr2**	41931095	41934089
**chr14**	3378623	3383033	**chr2**	52419916	52415465
**chr14**	3333123	3334754	**chr2**	41929019	41930761
**chr14**	3441504	3442726	**chr2**	52383745	52384973
**chr14**	3300945	3303443	**chr2**	41913983	41911476
**chr14**	3413416	3414888	**chr3**	29757116	29755631
**chr14**	3486907	3489362	**chr2**	52469759	52467079
**chr14**	3395993	3399477	**chr2**	52528039	52531578
**chr14**	3455265	3456970	**chr2**	52433232	52434962
**chr14**	3272821	3276743	**chr3**	29828150	29832203
**chr8**	17393659	17395191	**chr2**	52394521	52392991
**chr8**	17380362	17382152	**chr2**	52385537	52383744
**chr14**	3293189	3295276	**chr2**	41924526	41922427
**chr14**	3417476	3421124	**chr3**	29752759	29748883
**chr14**	3450846	3453140	**chr2**	52412415	52410062
**chr14**	3469221	3472060	**chr2**	52395855	52393013

Table S9 – Parameters used for each software program

Step	Tool	Parameters	Notes:
**a**			GCA_900406525.1 (fragmented), base assembly
**b**	MAC v2.0 (Tang et al. 2020)		Merged ABySS, SOAPdenovo2, MaSuRCA assemblies
**c**	LRSCAF v1.1.8 (Qin et al. 2019)	See [Suppl. Info]	Used with MaSuRCA super-reads, mapped with pblat (Wang & Kong 2019)
**d**	SCUBAT (https://github.com/elswob/SCUBAT)	-q=de_novo_transcriptome	Mapped with pblat
**e**	PEP_scaffolder (Zhu et al. 2016)	-q=AAs_from_de_novo_transcriptome	AAs predicted with TransDecoder
**f**	L_RNA_scaffolder (Xue et al. 2013)	-q=SRA:SRP046070	Mapped with pblat, 454 reads
**g**	P_RNA_scaffolder	-F=RNA_seq_forward_reads -R=RNA_seq_reverse_reads	Mapped with HISAT2 (--pen-noncansplice 1000000 -k 3 --rna-strandedness RF)
**h**	TGS-GapCloser (Xu et al. 2020)	--reads=MaSuRCA_super-reads --ne	
**i**	GMCloser (Kosugi et al. 2015)	--read_file=SRA:ERP108550 -l 100 -i 151 -d 40 --hetero	Paired Illumina reads from SRA archive used with published contigs [Suppl. Info]
**j**	Purge Haplotigs (Roach et al. 2018)	-l 2 -m 29 -h 190; -a 70	Mapped with minimap2 (Li 2018) (-ax sr); reads: SRA:ERP108550; See Suppl. Info for coverage histogram
**k**	RagTag (Alonge et al. 2022)	scaffold	Mapped with minimap2 (-ax asm10), haplotigs and published contigs used as reference
**l**	LRSCAF		
**m**	SCUBAT		
**n**	PEP_scaffolder		
**o**	L_RNA_scaffolder		
**p**	P_RNA_scaffolder		
**q**	TGS-GapCloser	--reads=haplotigs, published_contigs --ne	
**r**	Purge Haplotigs A70	-l 2 -m 25 -h 172; -a 70	See Suppl. Info for coverage histogram
**s**	RagTag	scaffold	Haplotigs from both j and r used as reference
**t**	LRSCAF		
**u**	SCUBAT		
**v**	PEP_scaffolder		

**w**	L_RNA_scaffolder		
**x**	P_RNA_scaffolder		
**y**	TGS-GapCloser	--reads=MaSuRCA_super-reads --ne	
**A**	Chromosemble	-t=GCA_024034495.1	C. laxa (n=6) used as reference; tool part of Satsuma (Grabherr et al. 2010)
**B**	LRSCAF		
**C**	PEP_scaffolder		
**D**	L_RNA_scaffolder		
**E**	P_RNA_scaffolder		
**F**	TGS-GapCloser	--reads=haplotigs(j),haplotigs(r), published_contigs –ne	Haplotigs from both j and r used as reference, published contigs
**Final**	GMCloser		

References

Alonge, M., Lebeigle, L., Kirsche, M., Jenike, K., Ou, S., Aganezov, S., Wang, X., Lippman, Z. B., Schatz, M. C., & Soyk, S. (2022). Automated assembly scaffolding using RagTag elevates a new tomato system for high-throughput genome editing. Genome Biology 2022 23:1, 23(1), 1–19. https://doi.org/10.1186/S13059-022-02823-7

Campbell, M. S., Holt, C., Moore, B., & Yandell, M. (2014). Genome Annotation and Curation Using MAKER and MAKER-P. Current Protocols in Bioinformatics, 48(1), 4.11.1-4.11.39. https://doi.org/10.1002/0471250953.BI0411S48

Kosugi, S., Hirakawa, H., & Tabata, S. (2015). GMcloser: closing gaps in assemblies accurately with a likelihood-based selection of contig or long-read alignments. Bioinformatics, 31(23), 3733–3741. https://doi.org/10.1093/BIOINFORMATICS/BTV465

Mohamadi, H., Khan, H., & Birol, I. (2017). ntCard: a streaming algorithm for cardinality estimation in genomics data. Bioinformatics, 33(9), 1324–1330. https://doi.org/10.1093/BIOINFORMATICS/BTW832

Qin, M., Wu, S., Li, A., Zhao, F., Feng, H., Ding, L., & Ruan, J. (2019). LRScaf: Improving draft genomes using long noisy reads. BMC Genomics, 20(1), 1–12. https://doi.org/10.1186/S12864-019-6337-2/TABLES/4

Roach, M. J., Schmidt, S. A., & Borneman, A. R. (2018). Purge Haplotigs: Allelic contig reassignment for third-gen diploid genome assemblies. BMC Bioinformatics, 19(1), 1–10. https://doi.org/10.1186/S12859-018-2485-7/FIGURES/5

Tang, L., Li, M., Wu, F. X., Pan, Y., & Wang, J. (2020). MAC: Merging Assemblies by Using Adjacency Algebraic Model and Classification. Frontiers in Genetics, 10, 506828. https://doi.org/10.3389/FGENE.2019.01396/BIBTEX

Xu, M., Guo, L., Gu, S., Wang, O., Zhang, R., Peters, B. A., Fan, G., Liu, X., Xu, X., Deng, L., & Zhang, Y. (2020). TGS-GapCloser: A fast and accurate gap closer for large genomes with low coverage of error-prone long reads. GigaScience, 9(9), 1–11. https://doi.org/10.1093/GIGASCIENCE/GIAA094

Xue, W., Li, J. T., Zhu, Y. P., Hou, G. Y., Kong, X. F., Kuang, Y. Y., & Sun, X. W. (2013). L_RNA_scaffolder: Scaffolding genomes with transcripts. BMC Genomics, 14(1), 1–14. https://doi.org/10.1186/1471-2164-14-604/FIGURES/6

Zhu, B. H., Song, Y. N., Xue, W., Xu, G. C., Xiao, J., Sun, M. Y., Sun, X. W., & Li, J. T. (2016). PEP-scaffolder: Using (homologous) proteins to scaffold genomes. Bioinformatics, 32(20), 3193–3195. https://doi.org/10.1093/BIOINFORMATICS/BTW378

Zimin, A. v., Marçais, G., Puiu, D., Roberts, M., Salzberg, S. L., & Yorke, J. A. (2013). The MaSuRCA genome assembler. Bioinformatics, 29(21), 2669–2677. https://doi.org/10.1093/BIOINFORMATICS/BTT476
