# Supplementary figures and images for: First draft reference genome and annotation of the alternative oil species Physaria fendleri
Source: G3 (Bethesda). 2024 May 28;14(9):jkae114. doi: 10.1093/g3journal/jkae114 (PMC11373644; doi:10.1093/g3journal/jkae114)

# Transcriptome Assembly Pipeline

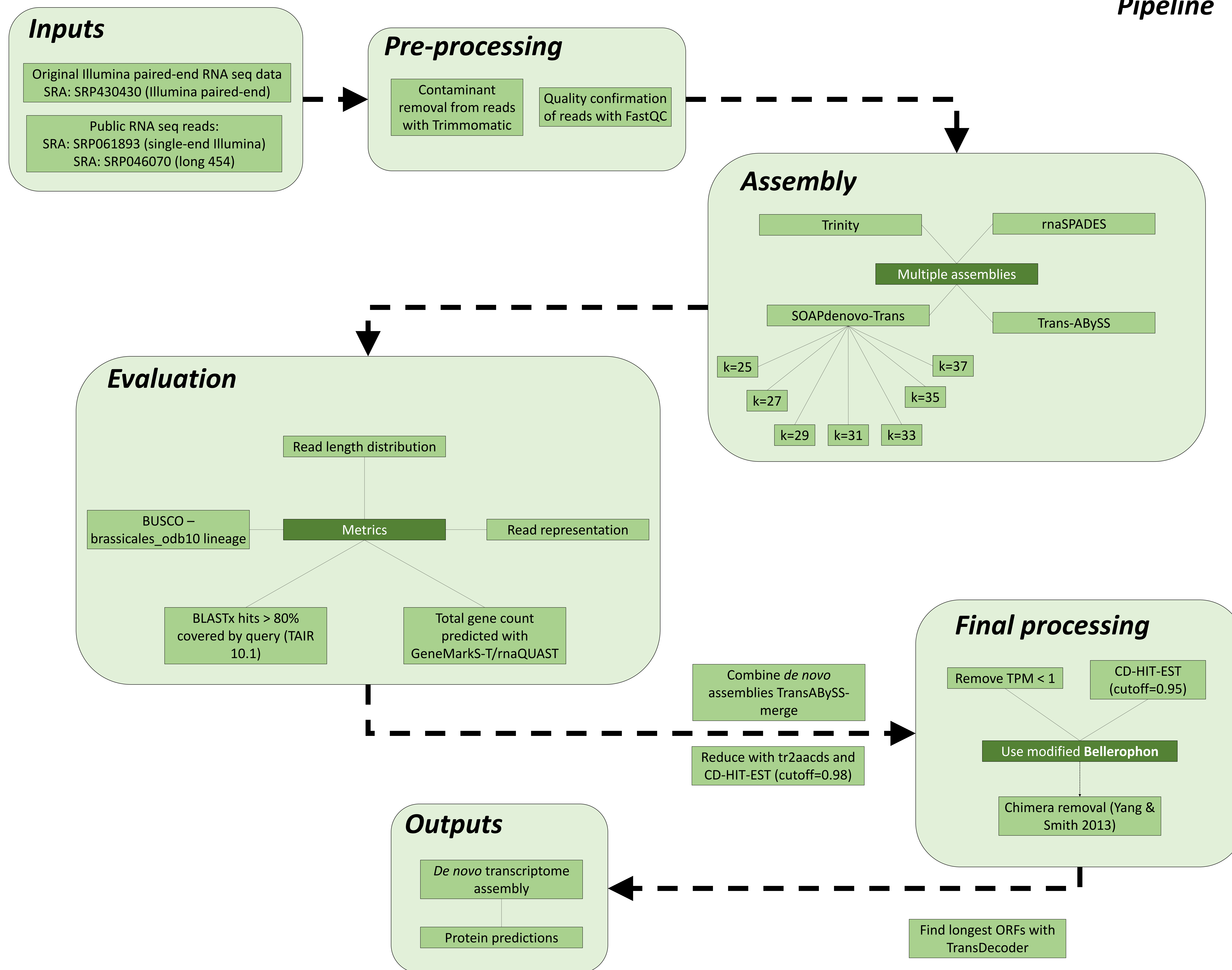

Supplement: jkae114_Supplementary_Data [file jkae114_supplementary_data.zip › Figure_S2_G3-2024-405031.pdf]

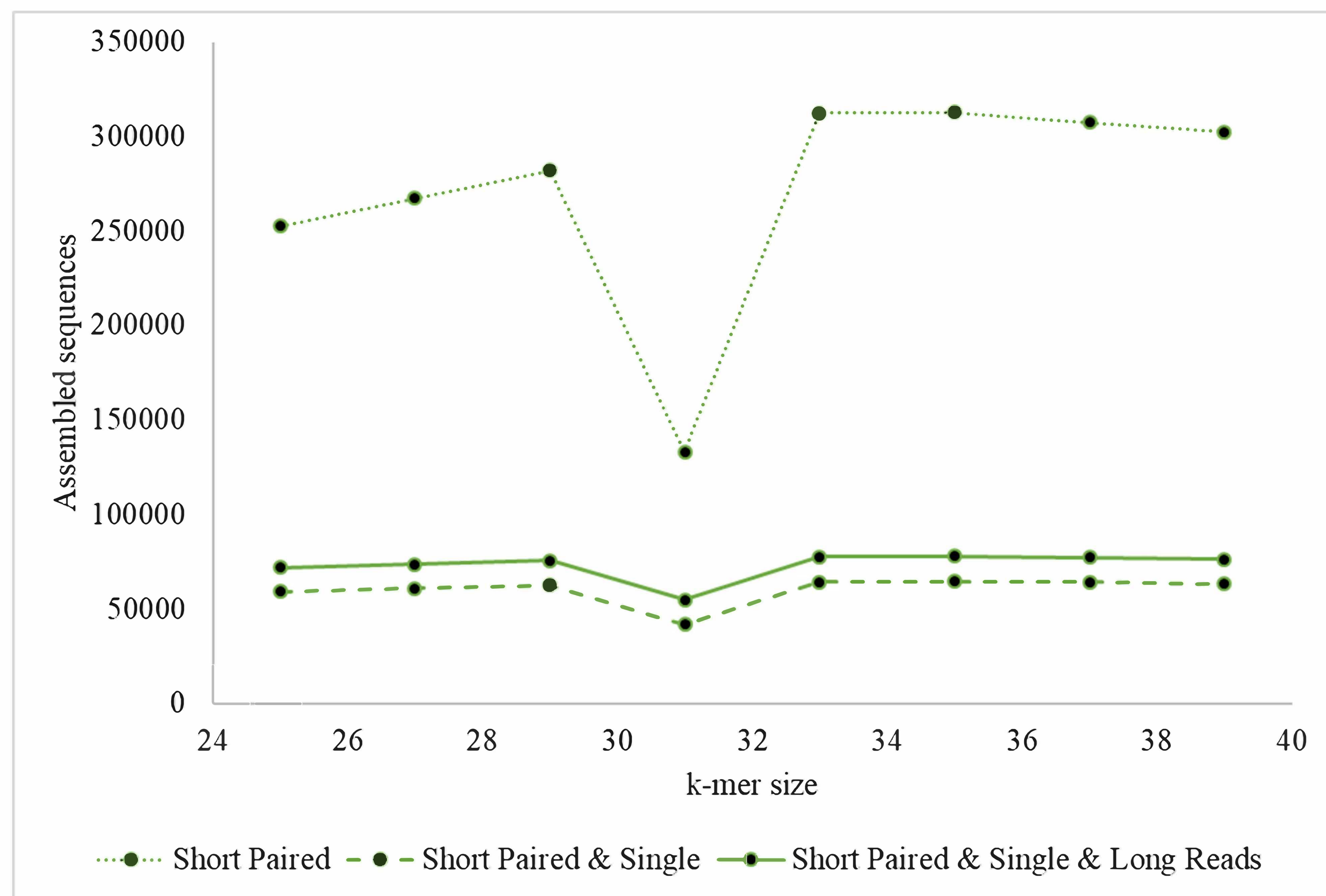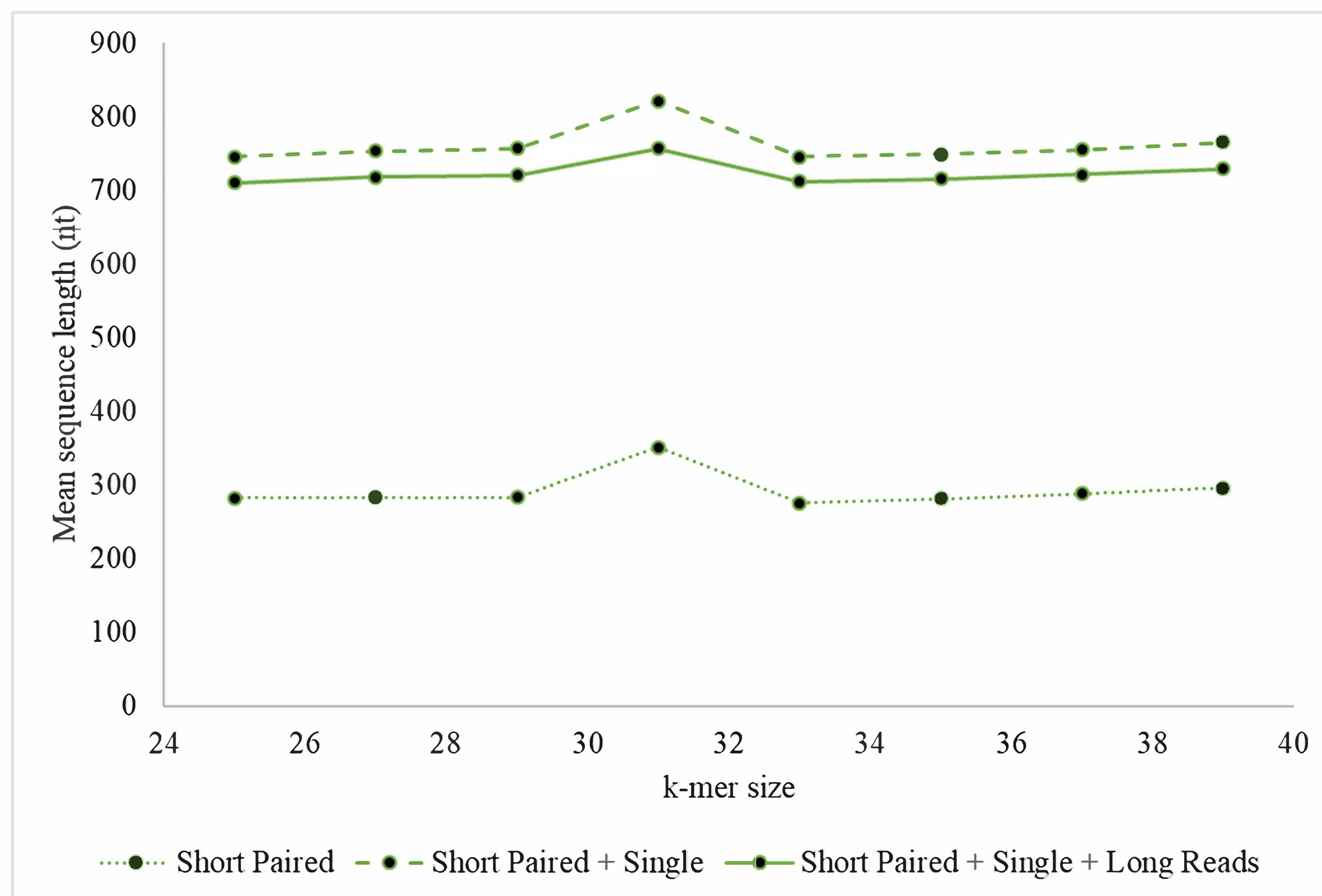

Supplement: jkae114_Supplementary_Data [file jkae114_supplementary_data.zip › Figure_S3_G3-2024-405031.pdf]

Genome Assembly Pipeline

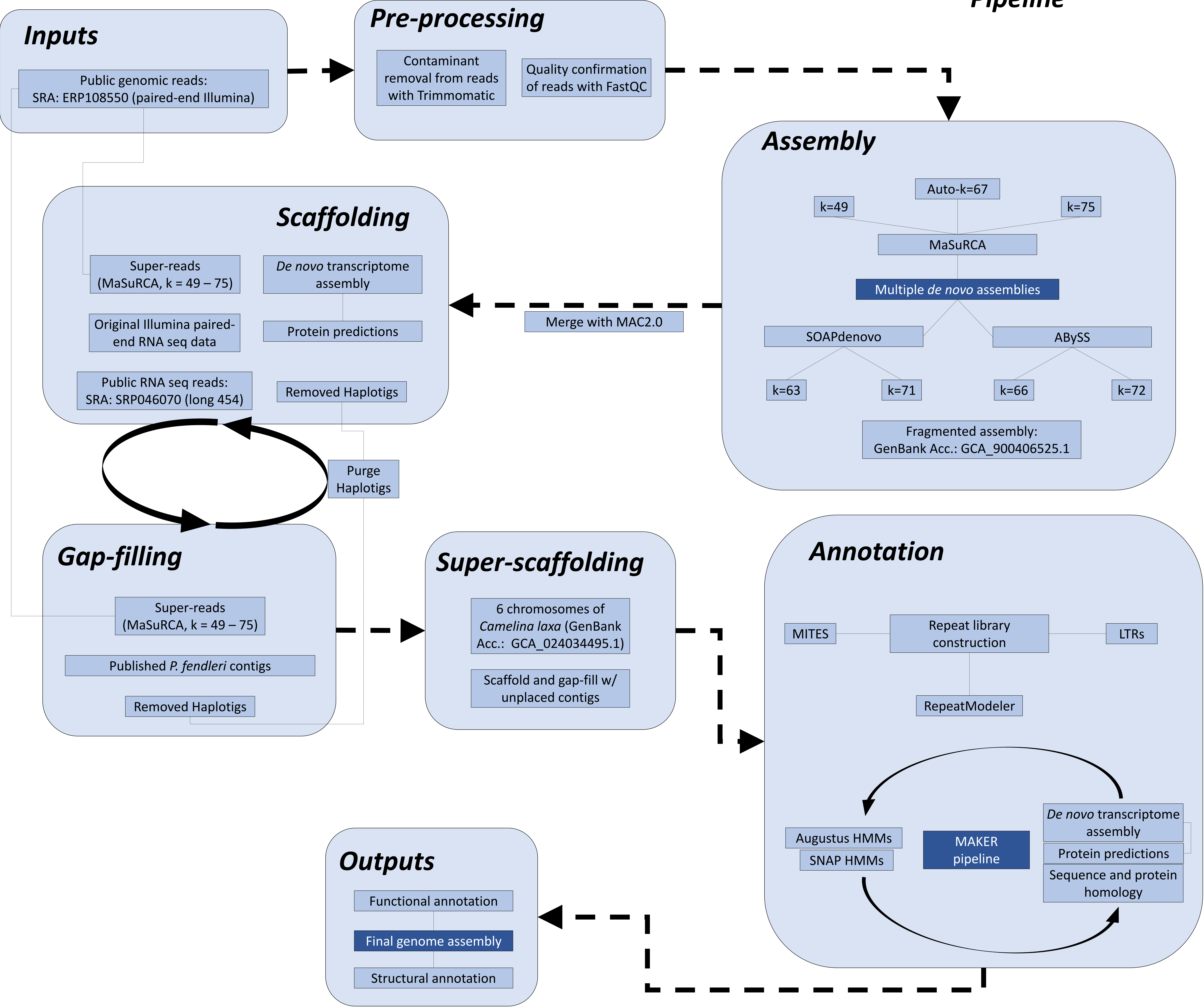

Supplement: jkae114_Supplementary_Data [file jkae114_supplementary_data.zip › Figure_S4_G3-2024-405031.pdf]

A

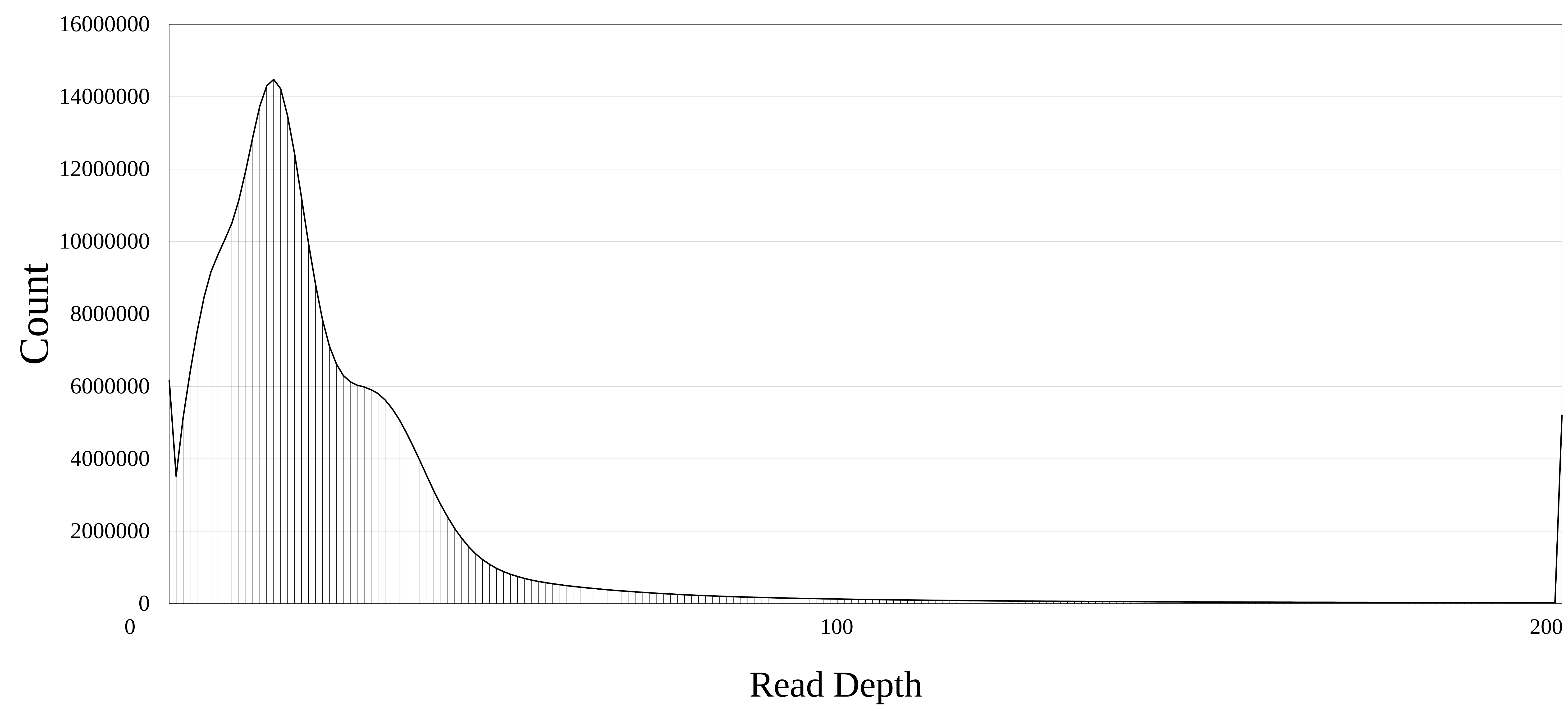

B

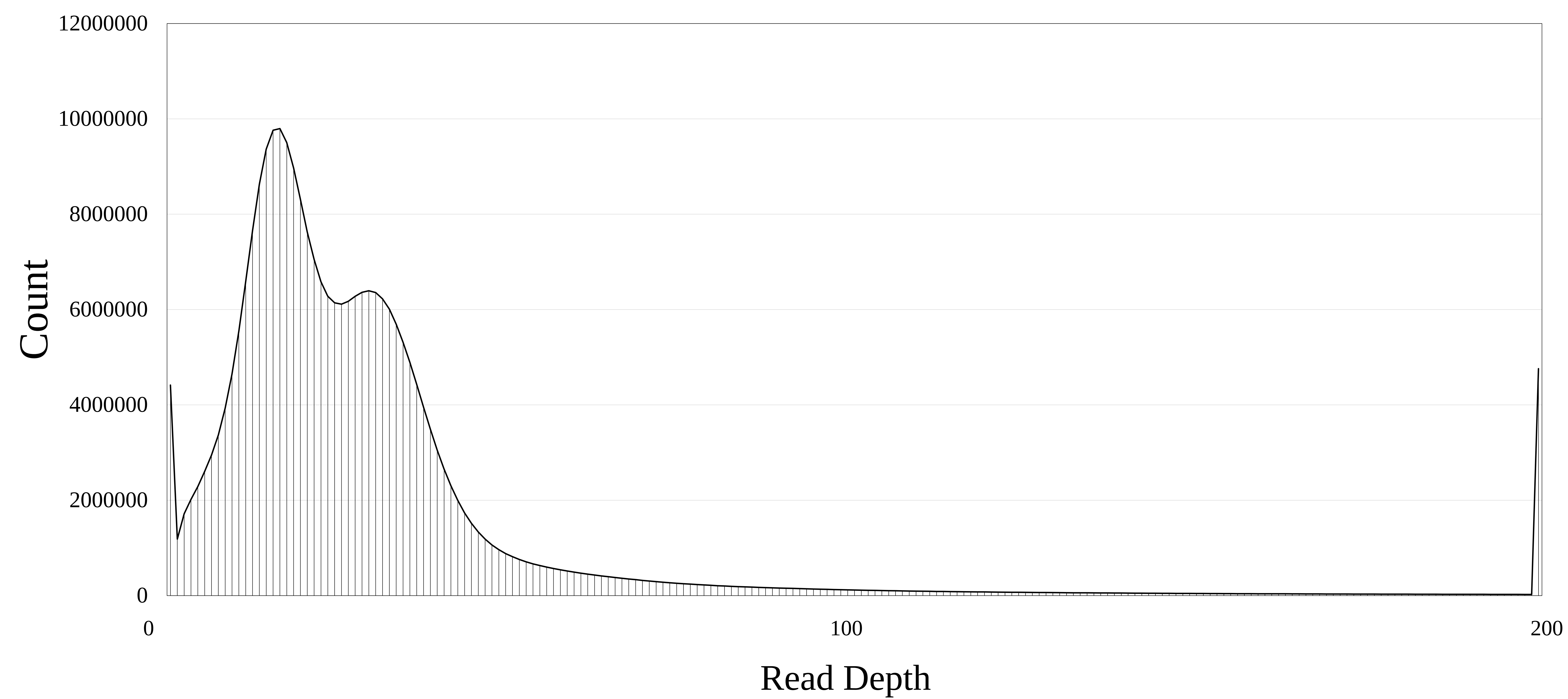

Supplement: jkae114_Supplementary_Data [file jkae114_supplementary_data.zip › Figure_S5_G3-2024-405031.pdf]
